# Mechanistic Model of Signaling Dynamics Across an Epithelial Mesenchymal Transition

**DOI:** 10.3389/fphys.2020.579117

**Published:** 2020-11-30

**Authors:** James D. Wade, Xiao-Kang Lun, Nevena Zivanovic, Eberhard O. Voit, Bernd Bodenmiller

**Affiliations:** ^1^Wallace H. Coulter Department of Biomedical Engineering, Georgia Institute of Technology, Emory University, Atlanta, GA, United States; ^2^Department of Quantitative Biomedicine, University of Zürich, Zürich, Switzerland; ^3^Institute of Molecular Life Sciences, University of Zürich, Zürich, Switzerland

**Keywords:** epithelial-to-mesenchymal transition (EMT), systems biology, ordinary differential equations, kinetic model, ERK pathway, AKT pathway, computational biology, single cell modeling

## Abstract

Intracellular signaling pathways are at the core of cellular information processing. The states of these pathways and their inputs determine signaling dynamics and drive cell function. Within a cancerous tumor, many combinations of cell states and microenvironments can lead to dramatic variations in responses to treatment. Network rewiring has been thought to underlie these context-dependent differences in signaling; however, from a biochemical standpoint, rewiring of signaling networks should not be a prerequisite for heterogeneity in responses to stimuli. Here we address this conundrum by analyzing an *in vitro* model of the epithelial mesenchymal transition (EMT), a biological program implicated in increased tumor invasiveness, heterogeneity, and drug resistance. We used mass cytometry to measure EGF signaling dynamics in the ERK and AKT signaling pathways before and after induction of EMT in Py2T murine breast cancer cells. Analysis of the data with standard network inference methods suggested EMT-dependent network rewiring. In contrast, use of a modeling approach that adequately accounts for single-cell variation demonstrated that a single reaction-based pathway model with constant structure and near-constant parameters is sufficient to represent differences in EGF signaling across EMT. This result indicates that rewiring of the signaling network is not necessary for heterogeneous responses to a signal and that unifying reaction-based models should be employed for characterization of signaling in heterogeneous environments, such as cancer.

## 1. Introduction

Intracellular signaling networks are biochemical systems that integrate spatio-temporal information regarding the intra- and extracellular environments into functional programs that drive cellular decisions (Dolmetsch et al., 1997; Kholodenko, 2006; Selimkhanov et al., 2014). Signals, such as extracellular ligand concentrations, are transduced by modulating the enzymatic activities and local concentrations of signaling mediators, such as kinases, within a cell. In traditional analysis, the structures of signaling networks are studied in biochemical experiments and are then formalized as graphs where nodes represent the activity state of signaling molecules and directed edges represent interactions between molecules. More recently, statistical modeling has been used to infer network structures in a data-driven manner (Sachs et al., 2005). In contrast to canonical representations of signaling networks as static structures, data-driven approaches of network inference have represented the structure of signaling networks as dependent upon context, including cell phenotype, type of input signal, and treatment, e.g., with an inhibitor, as well as unaccounted cell-to-cell variability (Hill et al., 2017; Petsalaki et al., 2015; Will and Helms, 2015; Brightman and Fell, 2000). The context-dependence of signaling is of particular consequence in diseases like cancer, where genetic errors lead to changes in the relative expression or function (Creixell et al., 2015) of signaling proteins and where the local microenvironment can be abnormal, resulting in signal responses that substantially differ from those of healthy cells (Altschuler and Wu, 2010). Consideration of signaling network rewiring between contexts has led to the development of novel treatment regimens (Lee et al., 2012). While useful, data-driven network inference requires large quantities of data and, typically, some prior knowledge of causative relationships. However, even though contexts are considered in this type of modeling, the results remain static representations of dynamic processes, which immediately limits predictions of cellular dynamics and responses (Kolitz and Lauffenburger, 2012).

Unlike graph-based network models, mechanistic models of signaling capture both the reaction network structure and the temporal dynamics of signaling, with residual noise attributed to natural cell-to-cell variability or stochasticity. These models consist of sets of reactions in the form of differential equations that describe how each signaling component changes over time as a function of the overall state of the system, and all model components and parameters have physical interpretations, such as concentrations, binding affinities, or reaction rates (Aldridge et al., 2006). Properly calibrated mechanistic models can be used to predict cellular dynamics from a snapshot of cell state and to analyze the consequences of observed or hypothetical alterations in relative concentrations or activities of components. The drawback of mechanistic models is that their construction relies on detailed prior knowledge of the reaction network structure and on multiple, targeted experiments that permit calibration of the model's parameter values (Aldridge et al., 2006; Kholodenko, 2006). The need for detailed prior information presents a great challenge when multiple contexts are to be considered (Halasz et al., 2016; Kholodenko, 2006).

Biochemical reasoning suggests that the signaling reaction structures themselves should be fixed, as the kinetics of a particular interaction does not change unless other factors or modulators are altered. This reasoning suggests that a mechanistic model should be “context-explicit,” reconciling the entire range of context-dependent signaling dynamics without network rewiring. In principle, therefore, it should be possible to construct a single mechanistic model that, combined with initial snapshots of individual cell states, explains and predicts signaling across many phenotypic contexts. In practice, however, the ability of a single mechanistic model to represent signaling across cell phenotypes is often limited due to ill-characterized differences in cellular milieu and gaps in knowledge, which are often considered as sources of natural stochasticity.

Here, we propose differential equation based mechanistic modeling—a model with static network structure—of single-cell mass cytometry data to investigate whether a context-dependent signaling network structure is necessary to represent differences in signaling dynamics in two distinct cell phenotypes: cells before and after an epithelial-mesenchymal transition (EMT). EMT is a developmental program through which epithelial cells trans-differentiate into a mesenchymal phenotype; EMT results in loss of cell-cell adhesion junctions, increased capacity for migration and invasion, and resistance to apoptosis (Fu et al., 2018). EMT has been implicated in the generation of metastatic and resistant cancer cell populations, and both bulk (Desai et al., 2015) and single-cell data (Krishnaswamy et al., 2018) have demonstrated EMT-associated alterations in signaling.

To study changes in signaling dynamics and network structure across an EMT, we use a previously established experimental model of TGF-β-induced EMT in Py2T murine breast cancer cells (Waldmeier et al., 2012). We performed mass cytometry to simultaneously quantify markers of epithelial and mesenchymal phenotypes, as well as the total expression and phosphorylation dynamics of multiple MAPK/ERK and PI3K/AKT signaling pathway components in response to stimulation with EGF, a proliferative signal that can drive tumor growth. These pathways and EGF signaling were chosen due to the robust signaling dynamics they provided in both epithelial and mesenchymal Py2T cell phenotypes.

To study the EMT-related changes in EGF signaling, we first applied a classical network-inference method to the data. This analysis suggested that the network structure of the ERK and AKT pathways is rewired in a phenotype-dependent manner. Then, using the same data and a mechanistic single-cell modeling approach based on the core principles of biochemical systems modeling (Savageau, 1976; Voit, 2000, 2013), we constructed, *ab initio*, two models of the signaling pathway, one for the mesenchymal phenotype and one for the epithelial phenotype. Intriguingly, accounting for unmeasured contextual variables allowed us to consolidate the two models into a single model with constant reaction structure and with very modest residual noise. This result presents proof-of-principle that rewiring does not occur during EMT but that alterations in signaling processes during this dramatic transition result from changes in the relative concentrations of signaling components. Our results suggest that further extending this type of single-cell model development, combined with highly-multiplexed single-cell measurements of intra- and extracellular states, has the potential to greatly reduce uncertainty that is otherwise attributed to stochasticity and to improve our ability to predict and analyze signaling responses in heterogeneous tissues and different disease contexts.

## 2. Results

**Figure 1:**
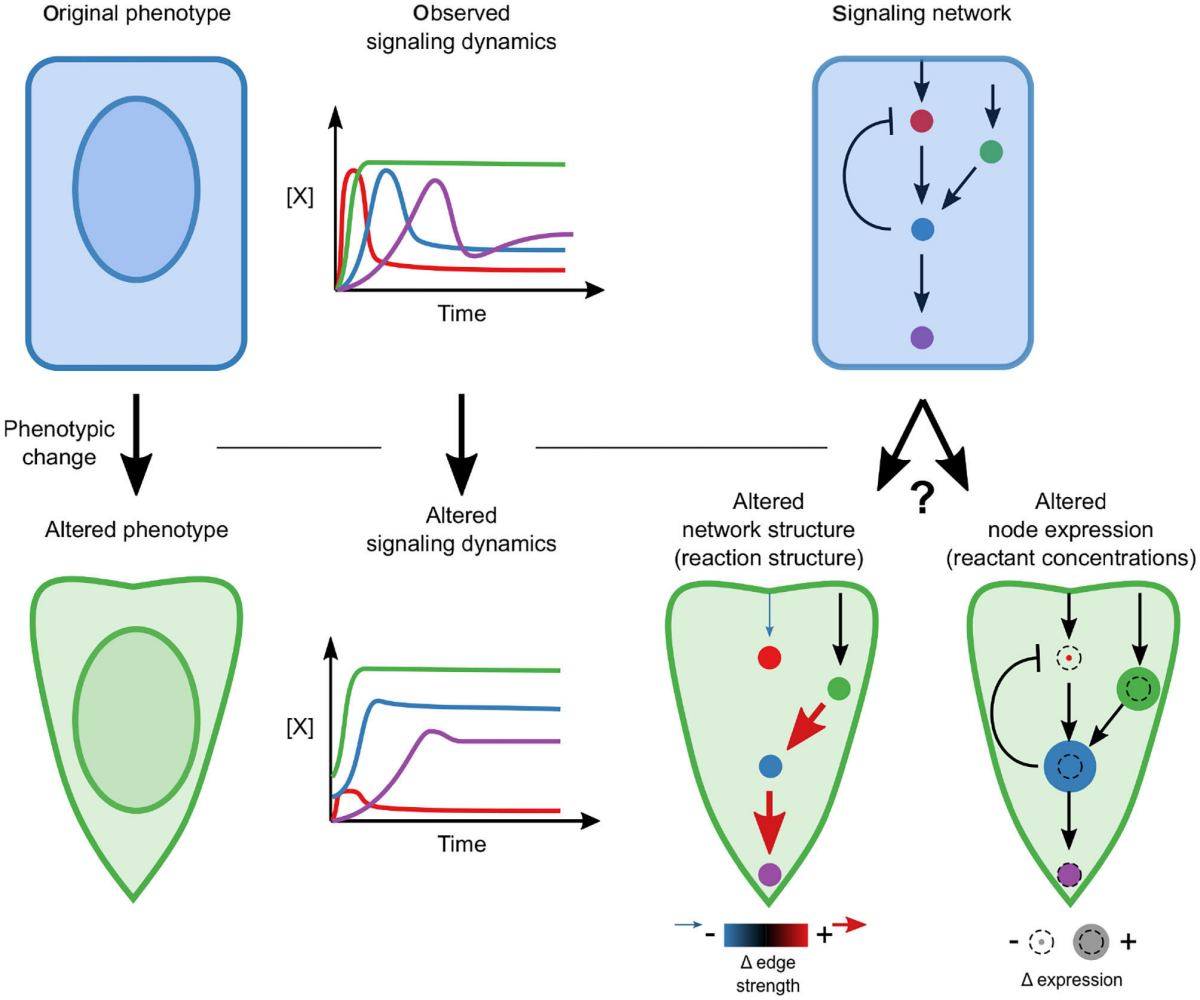
Conceptual overview. A phenotypic transition is associated with a changed signaling response. A key question is whether this alteration requires network rewiring (associated with the addition or elimination of edges) or whether the network structure is constant and moderate differences in relative concentrations drive differences.

During EMT, cells undergo dramatic changes in phenotype that are known to be driven by signaling; therefore, we sought to determine whether signaling networks are rewired or whether changes in the relative concentrations of signaling components are sufficient to permit altered dynamics (Figure 1). Specifically, we pose the hypothesis that a single, mechanistic model with fixed reaction network structure and kinetic parameters, calibrated with highly-multiplexed single-cell measurements of signaling protein state and expression, should be able to reconcile the differences in signaling across phenotypes.

To test our hypothesis, we used an experimental model of EMT, TGF-β-induced EMT in murine Py2T cells, and focused on EGF signaling dynamics in the proliferative, pro-growth, and pro-oncogenic MAPK/ERK and PI3K/AKT pathways (Wee and Wang, 2017). We used mass cytometry to quantify markers of epithelial and mesenchymal phenotypes and levels of phosphorylated and total signaling proteins. Specifically, we measured total and phosphorylated forms of MEK, ERK, and p90RSK (RSK) in the core ERK pathway, AKT and GSK3β in the AKT pathway, ribosomal protein S6 (S6), a downstream target of both ERK and AKT signaling, as well as cell cycle, death and size markers in 12 serial samples from a time course of EGF stimulation (for the full antibody panel, see Supplementary Table 4).

Our dataset contains 31-dimensional measurements across 34 conditions: Thirteen time points for EGF stimulation and four time points for unstimulated EGF control both before and after induction of EMT. Samples were analyzed in triplicate with an average of nearly 10,000 cells analyzed per sample.

### 2.1. Data-Driven Statistical Network Inference Suggests That the AKT/ERK Signaling Network Is Rewired in Response to TGF-β Treatment

**Figure 2:**
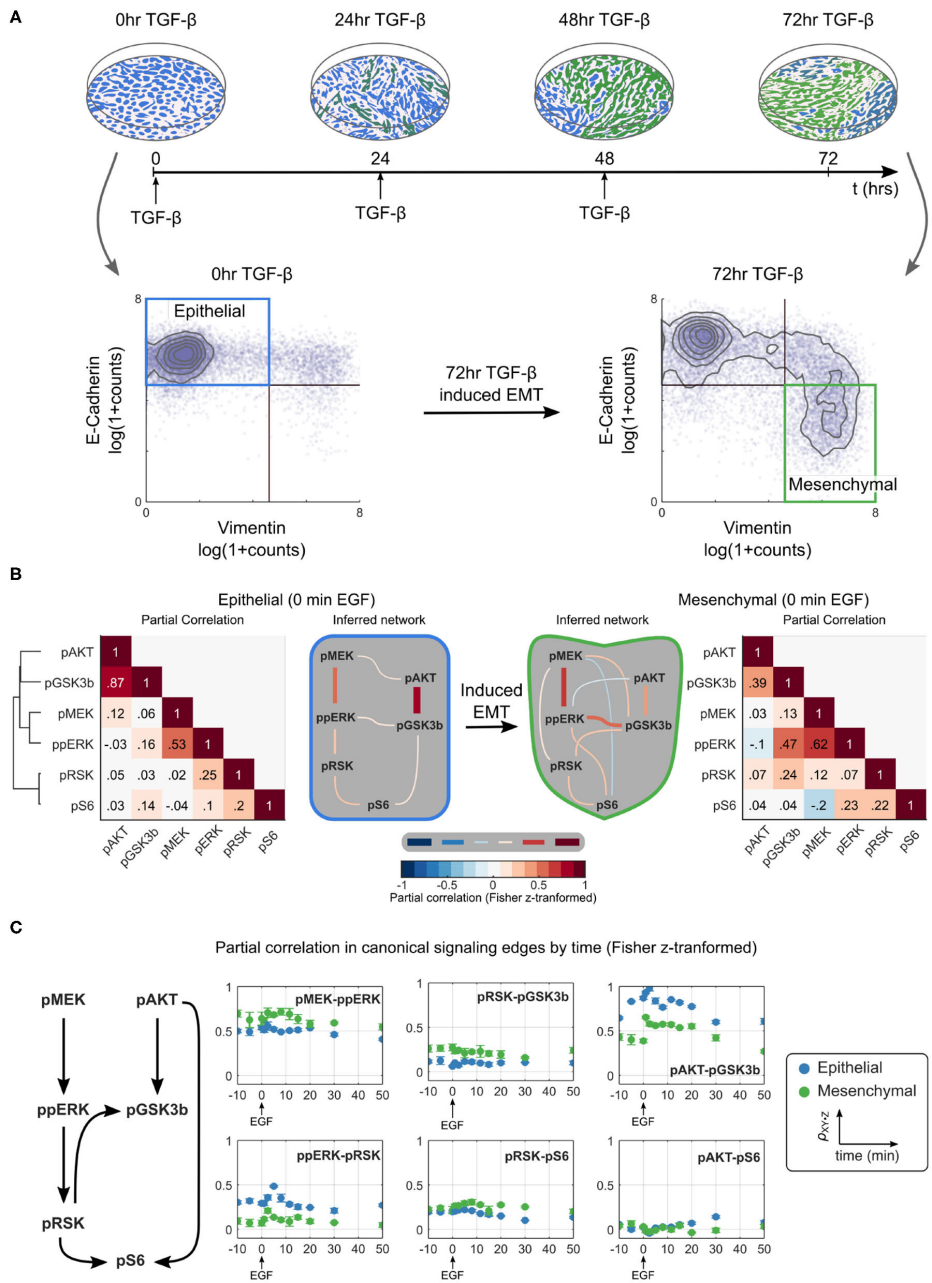
Partial correlation-based network inference suggests that the ERK/AKT signaling network is significantly rewired during EMT. **(A)** Overview of an EMT model (top) including an illustration of the visual phenotype and a molecular definition of epithelial and mesenchymal cells measured by mass cytometry at various time points (bottom). **(B)** Partial correlation (ρ_*XY*·*Z*_) analysis of phosphoproteins is used to infer the different network structures in epithelial and mesenchymal cells sampled across replicates. The threshold for accepting an edge between *X* and *Y* is defined as ρ_*XY*·**Z**_ ≥ |0.1|. All values are Fisher z-transformed. **(C)** Time dependence of signaling strength in canonical signaling edges. Blue and green represent epithelial and mesenchymal phenotypes, respectively. Error bars represent standard deviation of partial correlation across replicates.

To study signaling during EMT, we used a model of TGF-β induced EMT in Py2T cells (Waldmeier et al., 2012; Krishnaswamy et al., 2018). When grown in a monolayer, these cells are epithelial in visual and molecular phenotype with a homogeneous cobblestone appearance and high expression of the epithelial marker E-cadherin. Chronic treatment of cells with TGF-β causes them to transition to a mesenchymal phenotype; cells no longer grow in a monolayer, have an elongated shape, no longer express E-cadherin, and instead express mesenchymal markers, such as vimentin. As in previous studies, we defined epithelial cells as those with high levels of E-cadherin and low levels of vimentin expression. To generate a population of mesenchymal cells, samples were treated with TGF-β at 24 h intervals for 3 days; these cells expressed little E-cadherin and high levels of vimentin (Figure 2A; Supplementary Figure 1).

We first used traditional methods of statistical network inference to determine whether they would suggest that the ERK/AKT signaling network becomes rewired during EMT. Specifically, we used the data-driven approach of partial correlation analysis (Garmaroudi et al., 2010; Desai et al., 2015), which quantifies the correlation between two variables after removing potentially confounding correlations to other variables in the system. The partial correlation between all pairs of phosphoproteins in our panel for epithelial and mesenchymal cells is shown in Figure 2B (see Supplementary Figure 2 for the analysis including both phospho- and total proteins). Partial correlation values with magnitudes >0.1 were taken to define edges representing true interactions. In epithelial cells, partial correlation recovered the canonical pMEK-ppERK-pRSK-pS6 and pAKT-pGSK3β pathways (Mendoza et al., 2011; Manning and Cantley, 2007; Olayioye and Neve, 2000; Wee and Wang, 2017); note that GSK3β phosphorylation at S9 is inhibitory. By contrast, the canonical pAKT-pS6 (via p70S6 kinase) (Mendoza et al., 2011) and pRSK-pGSK3β (Manning and Cantley, 2007) relationships were attributed to pGSK3β-pS6 and ppERK-pGSK3β, respectively. The crosstalk suggested between pMEK and pAKT has not been reported previously, but could indirectly reflect known crosstalk between PI3K3CA and the RAF activator RAC (Ebi et al., 2013).

This analysis suggests that EMT rewired the signaling network. With the chosen cutoff, the mesenchymal cells appear to lose three (of seven) original edges and gain six new edges after the transition to mesenchymal cells. Compared to epithelial cells, in mesenchymal cells the edges ppERK-pRSK, pGSKβ-pS6, or pMEK-pAKT are lost and edges pMEK-pRSK, pMEK-pGSKβ, pMEK-pS6, ppERK-pAKT, ppERK-pS6, and pRSK-pGSKβ are gained. Noticeably absent in mesenchymal cells is a direct path from the AKT pathway to pS6. When different cutoffs were used, the specific edges gained and lost changed somewhat (see Supplementary Figure 2), but significant EMT-dependent rewiring was predicted at all cutoffs.

*Signaling is a dynamic process*. To assess the time dependence of various signaling states, we quantified the dynamics of network relationships by calculating the partial correlation between widely documented “canonical” network edges for each time point (Figure 2C). This analysis demonstrated that the strengths of most edge relationships, including that of the pMEK-ppERK edge, varied when compared before and after EMT. Such variation is expected, as existence of an edge can be interpreted as identification of signaling “activation.” Notably, at certain time points we observed two edges that were not observed at the steady state, the ppERK-pRSK edge in mesenchymal cells and the pAKT-pS6 edge in epithelial cells. The dependence of the network structure on context, such as time relative to stimulation and cell phenotype, illustrates that such a purely network-based statistical analysis, even if it is based on distributions of single-cell data, does not always accurately predict signaling responses.

### 2.2. Mechanistic Model With Constant Network Structure Permits Heterogeneity of Epithelial and Mesenchymal ERK/AKT Signaling

**Figure 3:**
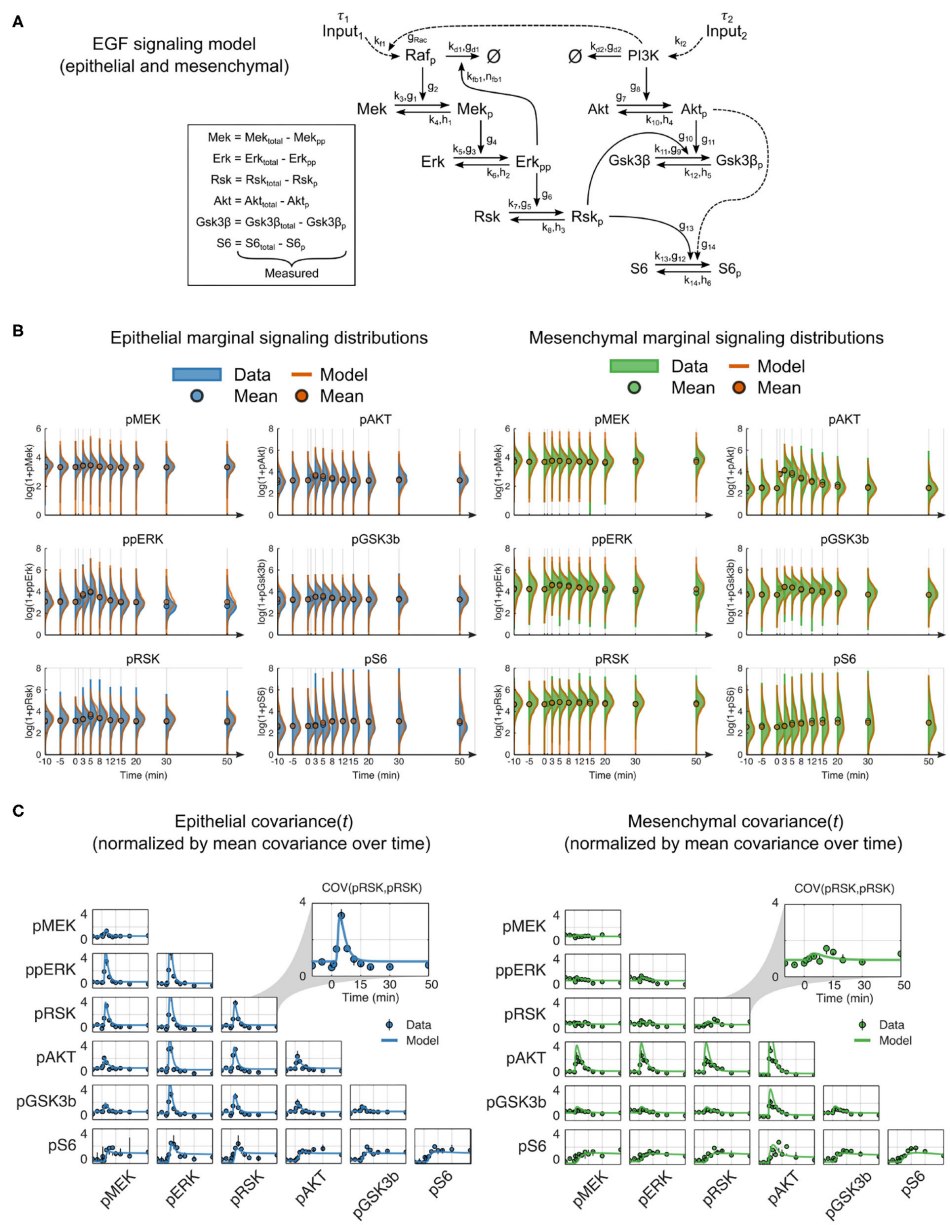
Mechanistic model of EGF signaling in ERK and AKT pathways with parameter fits to epithelial and mesenchymal cells. **(A)** Model reaction structure and measured variables used for each phenotype annotated with kinetic parameters. **(B)** Marginal distributions of (log_2_ transformed) data (solid) and model simulations (orange line) for dynamic signaling variables in epithelial (blue solid, left) and mesenchymal (green solid, right) cells. Circles represent means. To enhance visualization, the mean, rather than the marginal distribution, at the 1-min time point is not shown. **(C)** Data (symbols) and model simulations (line) of covariance between signaling variables over time in cells of both phenotypes. Black bars represent the range of covariances across replicates. All values in **(B,C)** were calculated by random subsampling of cells across experimental replicates.

We hypothesized that a dynamic mechanistic model with constant reaction network structure that includes context-dependent parameters should more accurately predict signaling dynamics in cells of both epithelial and mesenchymal phenotypes. Using prior knowledge combined with our multiplexed single-cell data, we constructed a reaction network model of the RAF-MEK-ERK-RSK-S6 and PI3K-AKT-GSK3β-S6 pathways (Mendoza et al., 2011; Manning and Cantley, 2007; Olayioye and Neve, 2000; Wee and Wang, 2017) that included pathway crosstalk at the level of PI3K to RAC (Ebi et al., 2013) and RSK to GSK3β (Manning and Cantley, 2007) (summarized in Figure 3A). The reactions were modeled in canonical format to reduce the inclusion of unmeasured reaction components, which is a streamlining step that has worked well in many other contexts (Savageau, 1976; Voit, 2000, 2013). We aggregated all upstream components into a single input with time delay (τ) and an unmeasured modifier variable, pRAF or PI3K, for each pathway. As they were not explicitly modeled, changes that occurred in upstream components (e.g., receptor expression and activation state) or regulation (e.g., altered membrane dynamics) during EMT were represented by changes in parameters, i.e., as magnitudes of pathway inputs.

In contrast to classical model calibration, which uses population averages for parameter estimation, we applied an approach that uses single-cell data to explicitly model single-cell variation and better constrains model parameters. This approach simulates sets of individual cells, where the initial state of each cell is taken from a snapshot measurement, and each combination of the individual cell trajectories represents an empirical multivariate distribution analogous to that derived from multiplexed single-cell measurements (see Methods).

After model fitting, simulations of independently subsampled cells showed strong agreement between model and data in both the marker distributions and parametric statistical features, such as the mean and covariance (Figures 3B,C, Supplementary Figure 3). Moreover, the shape of the model distributions fit overall very well; departing furthest from measurements in the density of pAKT for mesenchymal cells (Figure 3B). This deviation may be due to unmeasured reaction components or modulators that change during EMT, such as membrane-level variances due to cellular morphological switches. Overall, this result confirms that a constant reaction structure is sufficient to represent signaling across EMT.

### 2.3. Consolidation of ERK/AKT Signaling in Epithelial and Mesenchymal Cells Requires Only Minimal Adjustments of Mechanistic Model Parameters

**Figure 4:**
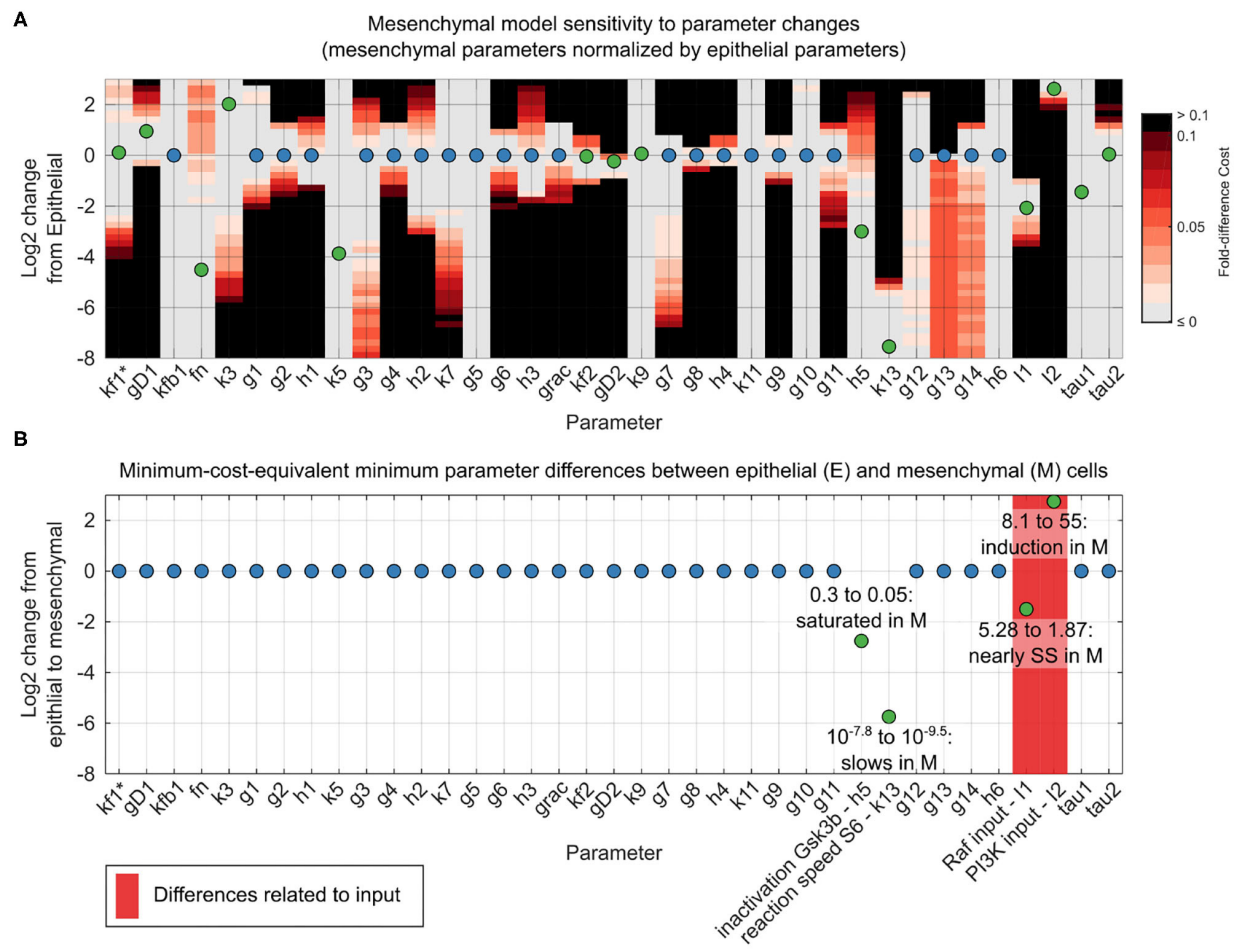
Reconciliation of mesenchymal and epithelial model parameters. **(A)** Sensitivity analysis of mesenchymal model parameter perturbations. Grid represents parameter changes on log_2_ scale of epithelial parameters used in Figure 3. Circles represent mesenchymal parameters used in Figure 3 (normalized to epithelial parameters in log_2_ scale). If epithelial and mesenchymal parameters are equal, the circles are blue; otherwise they are green. Grid color (color bar) represents percent increase in cost function for the mesenchymal cell model given a corresponding parameter change. **(B)** Minimal parameter difference between epithelial and mesenchymal parameter sets for constant mesenchymal function cost. Parameters associated with model inputs (input magnitude or degradation) are shown in red. Scale and circle colors are as in **(A)**.

Using our unifying reaction network structure, we investigated to what extent the parameters of the dynamic reaction model could be held constant across epithelial and mesenchymal cells. The original point estimates for the model parameter sets differed for the two phenotypes. However, for both phenotypes, there were ranges of parameter values within which the data fit very well. In most cases, these ranges overlapped between the epithelial and mesenchymal cell models. This quality of fit can be visualized in a grid-based sensitivity analysis of how individual parameter changes alter the quality of a model fit (Figure 4A). Although these results were encouraging, such univariate sensitivity analyses do not account for the potentially complex effects of changing multiple parameters simultaneously. We therefore systematically searched simultaneously for complete, close-by parameter sets that minimized the differences in parameters between epithelial and mesenchymal cells without decreasing model fitness in either context (Figures 4B,C).

As we had hypothesized, based on generic biochemical reasoning, analysis of the mechanistic model revealed that a constant network structure was sufficient to accurately predict signaling alterations during the transition from the epithelial to the mesenchymal state. Amazingly, of the 38 total model parameter values, only four required minor adjustments between epithelial and mesenchymal states: First, *I*_1_, the magnitude of the input to the ERK pathway, is 5.3 in the epithelial state and 1.9 in the mesenchymal state, approaching the pre-EGF steady-state value of 1, at which point there would be no stimulation of the ERK pathway. Second, *I*_2_, the magnitude of the input to the AKT pathway, increases by less than an order of magnitude from 8.1 in the epithelial state to 55 in the mesenchymal state. Third, parameter *h*_5_ decreased from 0.3 in the epithelial state to 0.05 in the mesenchymal state. This parameter is related to the sensitivity of pGSK3β dephosphorylation in response to increases in the concentration of pGSK3β, and accounted for a minute 1% change in model fitness. This change in *h*_5_ may not be surprising given the direct interpretation of the reaction moving from near zero-order (nearly saturated with respect to pGSK3β) to very near zero-order (even closer to saturation), combined with the fact that pGSK3β is generally higher in the mesenchymal state than the epithelial state at across all time points. Fourth, *k*_13_, which is related to the rate of S6 phosphorylation, decreases from 10^−7.8^ in the epithelial state to 10^−9.5^ in the mesenchymal state. This difference could be related to altered expression of unmonitored reaction components or to variations in local concentrations or diffusion rates. Importantly, almost all responses of the system could be explained without evoking natural stochasticity.

To assess the validity of model predicted changes to ERK and AKT pathway inputs, we used a previous molecular study of EMT-dependent signaling across multiple *in vitro* EMT models and more than 70 lung cancer cell lines (Salt et al., 2014). Our model prediction that the ERK pathway input, *I*_1_, decreases across EMT agreed with reported experimental findings that levels of phosphorylated EGFR, as well as total expression of other EGFR family members, were reduced in mesenchymal cell states compared to epithelial cell states. The other relevant prediction of our model, that the AKT pathway input *I*_2_ increased across EMT, also agreed with the experimental findings that PI3KCA, which encodes the catalytic p110α subunit of PI3K upstream of AKT activation, was upregulated and increased AKT pathway signaling across EMT. The consistency of these model predictions with independent experimental observations provides an orthogonal validation of the our model.

## 3. Discussion

Theoretically, differences in signaling responses across cells should be determined by relative differences in concentrations and states of reaction components, rather than by the idea of substantial reaction network rewiring. We show here that this is indeed the case for signaling in cells before and after the EMT in an *in vitro* model. To demonstrate this consistency, we performed multiplexed single-cell measurements and applied computational modeling to determine how EGF signaling in the ERK and AKT pathways changes with cell phenotype. We generated a large dataset; specifically, we used twice as many measured state variables as had been used previously to create a single-cell mechanistic model of signaling during EMT. Especially valuable for use with the mechanistic modeling approach was the inclusion of markers of total protein to evaluate variation in protein expression.

While our analysis focused on the response of the ERK and AKT pathways to EGF before and after EMT, we note that these two pathways, as well as the SMAD pathway, are involved in TGF-β-induced EMT itself. Furthermore, network-based analyses have shown that signaling via all three pathways is altered during in the same Py2T model of TGF-β-induced EMT used here (Krishnaswamy et al., 2018). Although our results suggest our mechanistic approach can be applied to characterize TGF-β signaling across EMT, our focus was on EGF signaling. The choice of EGF as the acute signaling stimulus over TGF-β was made, first, to avoid potentially confounding input signals due to the chronic TGF-β treatment used induce EMT, and second, due to the relevance of proliferative EGF signaling and potential in the tumor context.

We first used a traditional data-driven approach of network inference based on partial correlation, which suggested that the ERK/AKT signaling network had to be rewired in a phenotype-dependent manner during EMT, as was previously reported (Krishnaswamy et al., 2018). In stark contrast, the dynamic, single-cell mechanistic model with constant network structure and near constant kinetic parameter values, calibrated with state-of-the-art single-cell data, predicts that rewiring during EMT is not necessary. Instead, the mechanistic model reconciled the variation in signaling dynamics in epithelial cells vs. mesenchymal cells with only minor EMT-dependent adjustments to model parameters. In summary, a properly calibrated mechanistic model can represent signaling across a contextual change as large as EMT. This mechanistic model suggests that the hypothesis that rewiring of signaling networks does not occur during this phenotypic change is correct; rather cells modulate concentrations of reaction components to alter signaling.

The ability to explain variation in signaling responses from an individual snapshot of cell states is an exciting prospect, but we emphasize that it is necessary to, first, observe dynamics and, second, explicitly measure and model the primary sources of variation in order to obtain accurate predictions. The system components that affect the signaling dynamics will likely include not only levels or functional states of signaling proteins but also features of the microenvironment. For example, if a subpopulation of cells expressed a change of function mutation in a signaling protein, the new molecular species must be added to the model with its own associated kinetic parameters and the expression level must be measured or inferred. Thus, the main limitations of our approach are related to the ability to identify and measure the appropriate variables as well as to the computational cost of fitting mechanistic models to increasingly large systems. These limitations, however, will diminish as multiplexed single-cell measurement technologies and computational infrastructure improve.

Taken together, a single dynamic, mechanistic model with entirely constant reaction structure at the single-cell level can reconcile the EGF signaling dynamics of the ERK/AKT signaling network across an EMT. The only parameter differences we identified associated with the signaling responses of epithelial and mesenchymal phenotypes are slight alterations in four of the 38 total model parameters. Only two of the changes are related directly to kinetic parameters, while the other two are modest variations in the magnitudes of inputs to the pathway. These four EMT-dependent parameters can be interpreted as EMT-dependent changes to reaction components that are not explicitly measured; for example, the increase in *I*_2_ across EMT agrees with the report of an EMT dependent increase in PI3K upstream of AKT (Salt et al., 2014). Thus, we would expect the EMT-dependent variation in *I*_1_ and *I*_2_ to be reduced when including measurements of pEGFR and PI3K. Remarkably, accounting for upstream signaling changes that alter the magnitude of input signal (i.e., *I*_1_ and *I*_2_) to the ERK and AKT pathways was largely sufficient to explain EMT-dependent signaling dynamics, with the inferred EMT-dependent change in the S6 activation rate alone (*k*_13_) sufficient to reach at least 99% model fitness for both cell phenotypes.

Our analysis clearly demonstrates that the network structure of murine Py2T breast cancer cells is not rewired during EMT. No mechanisms or processes have to be added and none have to be removed. In fact, it appears that the signaling system is essentially deterministic. Our analysis suggests that the stochasticity inferred from previous studies is by and large a matter of unmeasured variables and a modest degree of intercellular variability. Given the ability of calibrated mechanistic models to analyze, simulate, and explain the dynamics from an initial snapshot of cell states, our data indicate that it is possible to account for cellular context via initialization of an appropriate model with multiplexed single-cell measurements. The proof-of-principle we present here represents an important step toward the construction of tumor-level dynamic models of signaling networks.

## 4. Methods

### 4.1. Cell Culture

Py2T cells were obtained from the laboratory of Gerhard Cristofori, University of Basel, Switzerland; their characterization was previously described (Waldmeier et al., 2012). Cells were tested for mycoplasma contamination upon arrival and regularly during culturing and before being used for experiments. Cells were cultured at 37°C in DMEM (D5671, Sigma Aldrich), supplemented with 10% FBS, 2 mM L-glutamine, 100 U/ml penicillin, and 100 μg/ml streptomycin, at 5% CO_2_. For cell passaging, cells were incubated with TrypLE^TM^ Select 10X (Life Technologies) in PBS in a 1:5 ratio (v/v) for 10 min at 37°C. For each experiment, cells were seeded at the density of 0.3 million cells per plate (100-mm diameter) and allowed to recover for 36 h.

### 4.2. TGF-β Stimulation

After reaching 60% confluence, cells were either mock-treated or treated with 4 ng/ml human recombinant TGF-β (Cell Signaling Technologies) for 72 h. Medium and TGF-β were replaced every 24 h until 24 h prior to EGF stimulation. For each condition, three biological replicates were cultured, harvested, and analyzed.

### 4.3. Cell Harvesting and EGF Stimulation

For cell harvest, cells were washed twice with PBS and incubated with TrypLE^TM^ Select 10X (Life Technologies) in PBS at a 1:5 ratio (v/v) for 10 min at 37°C. Following cell detachment, cells were mixed and resuspended in serum-free medium and allowed to recover from detachment for 2 h at 37°C and 5% CO_2_ with periodic shaking to avoid cluster formation. After the recovery period, samples were taken to establish baselines. EGF (Peprotech) was then added to a final concentration of 100 ng/ml. Samples were taken at {−10, −5, 0, 1, 3, 5, 8, 12, 15, 20, 30, 50} min relative to stimulation (*t* = 0) with EGF (the 0-min sample was not stimulated). At the time of sampling, paraformaldehyde (PFA, from Electron Microscopy Sciences) was added to the cell suspension to a final percentage of 1.6%, and cells were incubated at room temperature for 10 min. Crosslinked cells were washed twice with cell staining medium (CSM, PBS with 0.5% BSA, 0.02% NaN_3_). After centrifugation, ice-cold methanol was used to resuspend the cells, followed by a 10-min permeabilization on ice or long-term storage at −80°C.

### 4.4. Metal-Labeled Antibody Conjugation

The MaxPAR antibody conjugation kit (Fluidigm) was used to generate isotope-labeled antibodies using the manufacturer's standard protocol. After conjugation, the antibody yield was determined based on absorbance at 280 nm. Candor PBS Antibody Stabilization solution was used to dilute antibodies for long-term storage at 4°C.

### 4.5. Barcoding and Staining Protocol

Formalin-crosslinked and methanol-permeabilized cells were washed three times with CSM and once with PBS. Cells were incubated in PBS containing barcoding reagents (^102^Pd,^104^Pd,^105^Pd,^106^Pd,^108^Pd,^110^Pd,^113^In, and^115^In) at a final concentration of 100 nM for 30 min at room temperature and then washed three times with CSM (Bodenmiller et al., 2012). Barcoded cells were then pooled and stained with the metal-conjugated antibody mix (Supplementary Table 4) at room temperature for 1 h. The antibody mix was removed by washing cells three times with CSM and once with PBS. For DNA staining, iridium-containing intercalator (Fluidigm) diluted in PBS with 1.6% PFA was incubated with the cells at 4°C overnight. On the day of the measurement, the intercalator solution was removed, and cells were washed with CSM, PBS, and doubly distilled H_2_O. After the last washing step, cells were resuspended in doubly distilled H_2_O and filtered through a 70-μm strainer.

### 4.6. Mass Cytometry Analysis

EQ Four Element Calibration Beads (Fluidigm) were added to cell suspensions in a 1:10 ratio (v/v). Samples were analyzed on a Helios mass cytometer (Fluidigm). The manufacturer's standard operation procedures were used for acquisition at a cell rate of ~500 cells per second. After the acquisition, all .fcs files from the same barcoded sample were concatenated (Bodenmiller et al., 2012). Data were then normalized, and bead events were removed (Finch et al., 2013) before doublet removal and de-barcoding of cells into their corresponding wells using a doublet-filtering scheme and single-cell deconvolution algorithm (Zunder et al., 2015). Cytobank (http://www.cytobank.org/) was used for additional gating on the DNA channels (^191^Ir and^193^Ir) and^139^La/^141^Pr to remove remaining doublets, debris and contaminating particulates. Data were then exported as .fcs files for subsequent analysis.

### 4.7. Gating Epithelial and Mesenchymal Cells

Epithelial cells were gated as E-cadherin^*high*^/vimentin^*low*^ in samples without TGF-β treatment. Mesenchymal cells were gated as E-cadherin^*low*^/vimentin^*high*^ in samples treated for 3 days with TGF-β. Gating cutoffs are shown in Figure 2A. Treatment with TGF-β for longer than 3 days increases the percentage of Py2T population that undergoes EMT. Samples treated with TGF-β for 3 days also contained cells within the E-cadherin^*high*^/vimentin^*low*^ gate, but these were not considered contextually as epithelial cells.

### 4.8. Data Normalization and Scaling for Use in Modeling

Experimental measurements were normalized for comparisons across independent experiments and measurements. Cell events with >20 counts for cleaved PARP were removed as dead or apoptotic. Before using variables in modeling, they were linearly scaled to satisfy biological constraints, such as the total units of a protein must be greater than or equal to the units of the phosphorylated form. Cells used in fitting were subsampled across experimental replicates to reduce computational cost. A full description of normalization, scaling, and subsampling of data before use in modeling is presented in the Supplementary Material.

### 4.9. Partial Correlation-Based Network Inference

Given two random variables *X* and *Y* and a set of controlling variables **Z** = *Z*_1_, ..., *Z*_*n*_, the partial correlation ρ_*XY*·**Z**_ is a measure of the relationship between *X* and *Y* when the effects of the **Z** = *Z*_1_, ..., *Z*_*n*_ random variables have been accounted for. Mathematically, ρ_*XY*·**Z**_ is the correlation of the residuals *e*_*X*_ and *e*_*Y*_ that result from a linear regression of *X* and *Y* with **Z**, respectively.

To determine the cutoff for partial correlation-based network representations, thresholds were used to define a minimum *p*-value or correlation coefficient. In order to focus on stronger relationships, we used a threshold based on the partial correlation values. The choice of |ρ_*XY*·**Z**_| ≥ 0.1 as the threshold was made as a qualitative boundary between maximizing canonical and minimizing non-canonical signaling relationships in epithelial cells. Most notably, this setting captured the edge between the ERK pathway and pS6 as well as some form of crosstalk between the ERK and AKT pathways. The addition or subtraction of edges based on other thresholds may be readily calculated from the heatmaps provided in Figure 2, and in the context of both total and phosphoproteins, in Supplementary Figure 2. Supplementary Figure 2 illustrates the relationship between the partial correlation threshold and network edge number. Heatmap labels were ordered by hierarchical clustering the epithelial population values using single-linkage clustering and Euclidean distance. All partial correlations were calculated using log transformed data values of the form *log*(1 + *X*) .

### 4.10. Mechanistic Model of ERK/AKT Pathway Response to EGF

To model EGF signaling dynamics, we used the *Distribution-Free Single-Cell modeling* (DISCO) (Wade et al., 2020) approach described elsewhere in detail. Briefly, cells were assumed to have the same population-level kinetic parameters, which were determined by minimizing the maximum mean discrepancy (MMD) (Gretton et al., 2012), a statistical two-sample test of similarity for *n*-dimensional distributions, between simulated and experimentally measured distributions. Cell-to-cell variation in unmeasured components was captured in a subset of rate constants that were algebraically determined by a combination of model structure, population-level parameters, and steady-state measurements. To fit individual parameter sets for epithelial and mesenchymal cell populations, we subsampled 500–1,000 cells across the three replicates for each time point. Model equations may be found in the Supplementary Material.

### 4.11. Parameter Optimization

Parameter optimization was performed using a combination of global and local search methods. First, 50,000 initial parameter sets were sampled from a user-input parameter range (see Supplementary Table 5). Next, the 200 parameter sets associated with minimum cost were selected. Finally, each parameter set was refined through multiple rounds of optimization using the unconstrained local search algorithm fminsearch in the Matlab Optimization Toolbox (The MathWorks, Inc., Natick, MA). The fminsearch algorithm begins with a broad search perspective before focusing on a more precise local area. As regular re-initialization of the search improved results, each round of optimization was run for 300 iterations. If, at the end of a round, the cost value had improved by at least 2%, another round was initialized using the parameter set output by the algorithm. Otherwise, the optimization was terminated. After this local search approach, fminsearch was initialized a final time with the parameter set corresponding to the lowest cost among the 200 solutions and was run until the model cost stabilized. Point estimates of epithelial and mesenchymal model parameter sets are shown in Supplementary Table 5.

### 4.12. Sensitivity Analysis of Mechanistic Model Parameters

A grid-based sensitivity analysis was performed in the context of the population parameter vector Θ. The cells sampled for model fitting, either epithelial or mesenchymal, and the model structure were held constant. Given the best-fit point estimate of the parameter set for epithelial cells $\Theta_{E}^{*}=\theta_{E,1},...,\theta_{E,m}$, sensitivities were calculated as the change Δ in cost function *F* given a change Δ in the *j*th parameter θ_*E, j*_: $\frac{\Delta \text{F}}{\Delta \theta_{E,j}}$ where Δθ_*E, j*_ was defined using a log_2_ fold-change of increments of 0.25 in the range of [−8, 3] when applied to the best parameter $\theta_{E,j}^{*}$ for epithelial cells. Notably, this definition implies that parameter sensitivities for the epithelial and mesenchymal models were calculated using the same set of values.

### 4.13. Reconciliation of Mechanistic Model Parameters Across EMT

Reconciliation between the best points estimates for epithelial and mesenchymal population parameter sets $\Theta_{E}^{*}$ and $\Theta_{M}^{*}$, respectively, was performed by finding the minimum difference between all corresponding population parameters θ_*E*_ and θ_*M*_ that did not increase the cost *F* associated with either best-fit parameter set:

(1) $$\begin{array}{l} \left\{{\hat{\Theta }}_{E^{*},j},{\hat{\Theta }}_{M^{*},j}\right\}=\min_{j}|\Theta_{E,j}^{*}-\Theta_{M,j}^{*}|\\ \text{ subject to}\\ \;F\left({\hat{\Theta }}_{E^{*}}\right)\;\leq F\left(\Theta_{E}^{*}\right)\\ \;F\left({\hat{\Theta }}_{M^{*}}\right)\;\leq F\left(\Theta_{M}^{*}\right) \end{array}$$

Point estimates of epithelial and mesenchymal model parameters after reconciliation (${\hat{\Theta }}_{E^{*}},{\hat{\Theta }}_{M^{*}}$) are shown in Supplementary Table 5.

## Data Availability Statement

The data set with raw and gated .fcs files and code including model implementation in Matlab and Matlab workspace variables used for modeling are available from Mendeley data: doi: 10.17632/c9h4rx5sf9.

## Author Contributions

JW, BB, and EV conceptualized the study and wrote the manuscript. JW, X-KL, and NZ designed and performed the experiments. JW conceptualized and performed the analysis. All authors reviewed and edited the final manuscript.

## Conflict of Interest

The authors declare that the research was conducted in the absence of any commercial or financial relationships that could be construed as a potential conflict of interest.
