# Supplementary material for: Mechanistic Model of Signaling Dynamics Across an Epithelial Mesenchymal Transition
Source: Front Physiol. 2020 Nov 30;11:579117. doi: 10.3389/fphys.2020.579117 (PMC7733964; doi:10.3389/fphys.2020.579117)
Supplement: Supplementary file 1 [file Data_Sheet_1.PDF]

Supplementary Notes: Mechanistic Model of Signaling Dynamics Across an Epithelial Mesenchymal Transition

James D. Wade, Xiao-Kang Lun, Nevena Zivanovic, Eberhard O. Voit, Bernd Bodenmiller

Introduction

Here, we provide additional details regarding the single-cell ODE model of EGF signaling as presented in the main text. This material includes description of data exclusion and scaling before use in modeling, the mathematical formulation of the model, and supplementary figures and tables.

Data exclusion criteria

Antibody labeling and cell staining were optimized to minimize the number of cell events with zero or low ion counts in measurement channels used for modeling. Furthermore, measured cell events with fewer than 5 ion counts in total protein channels were excluded. The choice of five counts was made as a trade-off between the number of discarded events, which increases as the threshold increases, and the uncertainty of single-cell measurements, which is a decreasing function of ion counts. This cutoff excluded fewer than 1% of cells per sample.

Data scaling for use in modeling

Mass cytometry provides relative values of protein abundance. Absolute values depend on factors such as antibody labeling efficiency, antibody staining, and detection sensitivity. Although relative differences between measured protein abundances can often be subsumed into reaction parameters, we rescaled measured protein levels by measurement channel. As absolute values of protein abundance were not available for Py2T cells, total protein abundance values were taken to be 500 in arbitrary units. These values were used to estimate the average concentration $\bar{\mathbf{x}}_j$ of each protein j in a cell. Protein measurements were then linearly scaled as follows:

Given a protein j, its associated average concentration $\bar{\mathbf{x}}_j$ in a sample and its measured steady-state distribution $D_{\mathbf{x}_{j|ss}}$, a scaling factor z_j was calculated as

$$z_j = \frac{\bar{\mathbf{x}}_j}{\mathrm{E}\big[D_{\mathbf{x}_{j|ss}}\big]}. \tag{1}$$

This implementation represents a linear scaling factor of the experimentally measured steady-state distribution of protein j such that the mean of the steady-state distribution $\mathrm{E}[D_{\mathbf{x}_{j|ss}}]$ is equal to the population average $\bar{\mathbf{x}}_j$, arbitrarily set at 500. For all experimental measurements, each total protein j was scaled using the corresponding z_j. In other words, all time points were scaled using a factor calculated at steady state.

Steady-state levels of phosphoproteins clearly cannot be greater than those of the corresponding total protein. Additionally, if steady-state phosphoprotein levels are too high relative to total protein, the relative increase in phosphorylation levels in the model would be capped due to a lack of unphosphorylated protein. To avoid these cases, phosphoprotein distributions were scaled such that the average steady-state value of a phosphoprotein was a fractional value of the total protein level. These additional scaling factors for phophoproteins relative to total proteins were set to 0.06; i.e., initial phosphoprotein levels were, on average, 6% of total protein. These values were chosen to reduce the number of cells in violation of the "active cannot be greater than total" constraint at steady state. In the rare case that individual cells violated this constraint, these cells were excluded from the sample for analysis.

Model of EGF signaling in the MAPK/ERK cascade

In this section, we describe the mathematical formulation of our model of EGF signaling in the MAPK/ERK cascade.

State variables

The model uses eight state variables to describe changes in ERK pathway signaling components.

State variable	Description
I_1	Input to RAF
pRaf	Active RAF
Mek	Inactive MEK
pMek	Active MEK
Erk	Inactive ERK
ppErk	Active ERK
Rsk	Inactive RSK
pRsk	Active RSK
I_2	Input to PI3K
PI3K	Active PI3K
Akt	Inactive AKT
pAkt	Active AKT
pGsk3b	Inactive GSK3β
Gsk3b	Active GSK3β
S6	Inactive S6
pS6	Active S6

Table S1: ODE model state variables

Active and total MEK, ERK, RSK, S6, AKT, and GSK3β were measured. After scaling, inactive forms were calculated as Inactive = Total − Active. The inputs I_1 and I_2 and initial values of pRAF and PI3K were assumed to be the same for all cells. These choices were made to reduce prior assumptions. The variation in these components that were not explicitly measured were captured by the single-cell parameters k_{d1}, k_4, k_{d2}, and k_{10}, which were obtained from steady-state measurements.

Kinetic parameters

Parameter	Description
k_{f1}	Activation rate constant of pRaf by Input_1
g_{d1}	Kinetic order of pRaf inactivation
k_{fb1}	Hill function inflection point of negative feedback from ppErk
f_n	Hill coefficient of negative feedback from ppErk
k_3	Activation rate constant of pMek
g_1	Kinetic order of pMek activation by Mek
g_2	Kinetic order of pMek activation by pRAF
h_1	Kinetic order of pMek inactivation by pMek
k_5	Activation rate constant of ppErk
g_3	Kinetic order of ppErk activation by Erk
g_4	Kinetic order of ppErk activation from pMek
h_2	Kinetic order of ppErk inactivation from ppErk
k_7	Activation rate constant of pRsk
g_5	Kinetic order of Rsk activation by Rsk
g_6	Kinetic order of Rsk activation by ppErk
h_3	Kinetic order of pRsk inactivation by pRsk
g_{Rac} k_{f2}	Activation rate constant of PI3K by Input_2
g_{d2}	Degradation rate constant of PI3K signal
k_9	Activation rate constant of pAkt
g_7	Kinetic order of pAkt activation by Akt
g_8	Kinetic order of pAkt activation by PI3K
h_4	Kinetic order of pAkt inactivation by pAkt
k_{11}	Inctivation rate constant of Gsk3b
g_9	Kinetic order of pGsk3b inactivation by Gsk3b
g_{10}	Kinetic order of pGsk3b inactivation by pRsk crosstalk
g_{11}	Kinetic order of pGsk3b inactivation by pAkt
h_5	Kinetic order of pGsk3b activation by pGsk3b
k_{13}	Activation rate constant of pS6
g_{12}	Kinetic order of pS6 activation by S6
g_{13}	Kinetic order of pS6 activation by pRsk
g_{14}	Kinetic order of pS6 activation by pAkt
h_6	Kinetic order of pS6 inactivation by pS6

Table S2: Model parameters

The rate constants $\{k_d1, k_4.k_6, k_8, k_{d2}, k_{10}, k_{12}, k_{14}\}$ were collected in $\boldsymbol{\Phi_k}$ and computed from the steady-state equations. All other parameters were collected in $\boldsymbol{\Theta}$ and, therefore, equal across all cells and used as decision variables in the optimization algorithms.

Inputs

The ERK/AKT pathway components considered in our model were in a pseudo-steady state at time scale of our experiments (1 hour). Thus, our model must also be at steady state before addition of EGF. We arbitrarily chose the input value of $I_1 = I_2 = I_{ss} = 1$ as the pre-stimulation steady-state inputs to our model. We used the time delays τ_1 and τ_2 to represent the delay between experimental

Input	Description
I_1	Input (EGF) to pRaf
I_2	Input (EGF) to PI3K
I_{ss}	Input at steady state (before EGF addition: $t < \tau$)
τ_1	Time delay between addition of EGF and initial pRaf signaling
τ_2	Time delay between addition of EGF and initial PI3K signaling

Table S3: Model inputs

addition of EGF to the medium and the time when the signal reached the ERK and AKT signaling branches, respectively. In other words, τ_1 represents the time it takes for signal to be transmitted by all relevant reactions including receptor-ligand binding, receptor activation, and RAF activation. Similarly, τ_2 represents the time required for PI3K activation. Although the exact values of τ_1 and τ_2 for each cell are undoubtedly variable across the population, as not all cells will encounter the EGF signal at the same moment, we assumed that the delay time (τ_1 and τ_2) was the same for each cell; this maintained the entirely deterministic nature of our model. Thus, in the model for each $i \in \{1, 2\}$, if time $t < \tau_i$, then Input $I = I_{ss} = 1$. Once time $t = \tau_i$, then input is reset to represent EGF addition and Input $I = I_i$. At all times $t \geq \tau_i$, input I_i is a dependent variable and determined by solution of the ODE system.

Model equations

$$
\begin{aligned}
\dot{I_1} &= -k_{f1} * (I_1 - I_{ss}) \\
p\dot{Raf} &= k_{f1} * I_1 * PI3K^{g_{Rac}} - k_{d1} * pRaf^{g_{D1}} * \frac{ppErk^{f_n}}{k_{fb1}^{f_n} + ppErk^{f_n}} \\
\dot{Mek} &= -k_3 * Mek^{g_1} * pRaf^{g_2} + k_4 * pMek^{h_1} \\
p\dot{Mek} &= k_3 * Mek^{g_1} * pRaf^{g_2} - k_4 * pMek^{h_1} \\
\dot{Erk} &= -k_5 * Erk^{g_3} * pMek^{g_4} + k_6 * ppErk^{h_2} \\
pp\dot{Erk} &= k_5 * Erk^{g_3} * pMek^{g_4} - k_6 * ppErk^{h_2} \\
\dot{Rsk} &= -k_7 * Rsk^{g_5} * ppErk^{g_6} + k_8 * pRsk^{h_3} \\
p\dot{Rsk} &= k_7 * Rsk^{g_5} * ppErk^{g_6} - k_8 * pRsk^{h_3} \\
\dot{I_2} &= -k_{f2} * (I_2 - I_{ss}) \\
\dot{PI3K} &= k_{f2} * I_2 - k_{d2} * pPI3K^{g_{D2}} \\
\dot{Akt} &= -k_9 * Akt^{g_7} * PI3K^{g_8} + k_{10} * pAkt^{h_4} \\
p\dot{Akt} &= k_9 * Akt^{g_7} * PI3K^{g_8} - k_{10} * pAkt^{h_4} \\
\dot{Gsk3b} &= -k_{11} * Gsk3b_{g_9} * pRsk^{g_{10}} * pAkt^{g_{11}} + k_{12} * pGsk3b^{h_5} \\
p\dot{Gsk3b} &= k_{11} * Gsk3b_{g_9} * pRsk^{g_{10}} * pAkt^{g_{11}} - k_{12} * pGsk3b^{h_5} \\
\dot{S6} &= -k_{13} * S6^{g_{12}} * pRsk^{g_{13}} * pAkt^{g_{14}} + k_{14} * pS6^{h_6} \\
p\dot{S6} &= k_{13} * S6^{g_{12}} * pRsk^{g_{13}} * pAkt^{g_{14}} - k_{14} * pS6^{h_6}
\end{aligned}
$$

Supplementary Figures and Tables

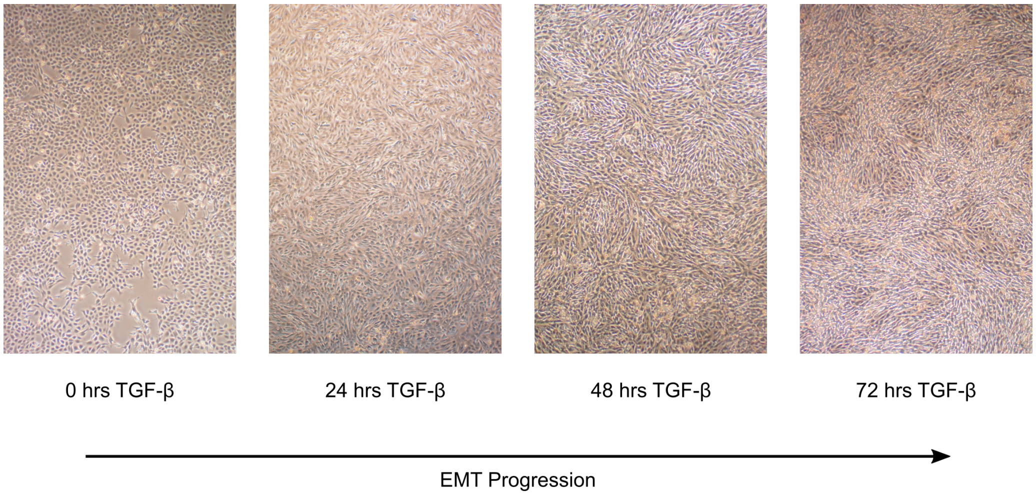

Figure S1: Microscopy images of TGF-β treatment inducing an EMT. At day 0 all cells grow in a monolayer with a cobblestone epithelial morphology. By day 3, some of the cells are no longer constrained to monolayer growth and have transitioned to an elongated mesenchymal morphology.

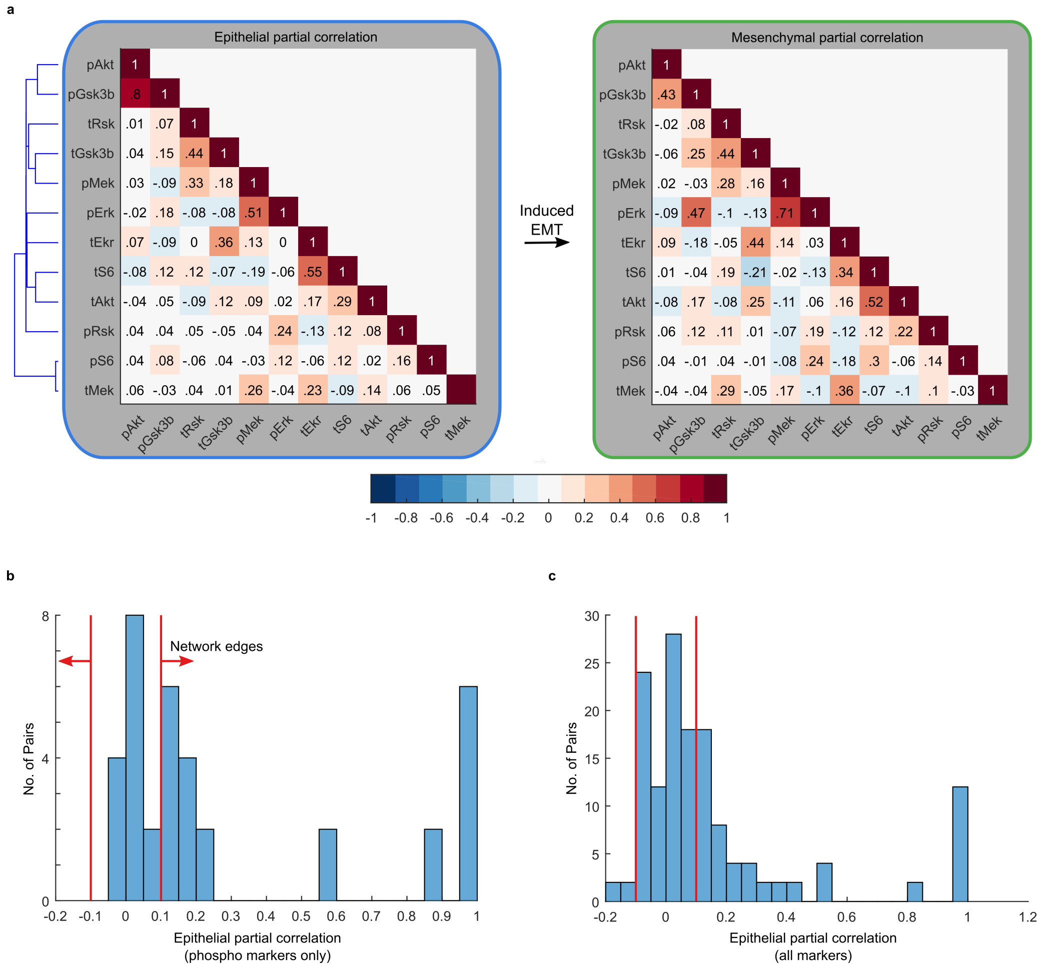

Figure S2: Partial correlation of phospho- and total signaling kinases for epithelial and mesenchymal cell populations. (**a**) Heatmaps of correlations of phospho- and total kinases ordered by clustering of the partial correlations of epithelial cells as shown in the dendrogram. Partial correlations were Fisher z-transformed. (**b**) Partial correlation values for epithelial cells as in Figure 2b. The cutoffs for network edges of $\rho \geq |0.1|$ are marked by the red lines. (**c**) The distribution of partial correlation values for epithelial cells as in Figure S2a. Red lines indicate $\rho = |0.1|$.

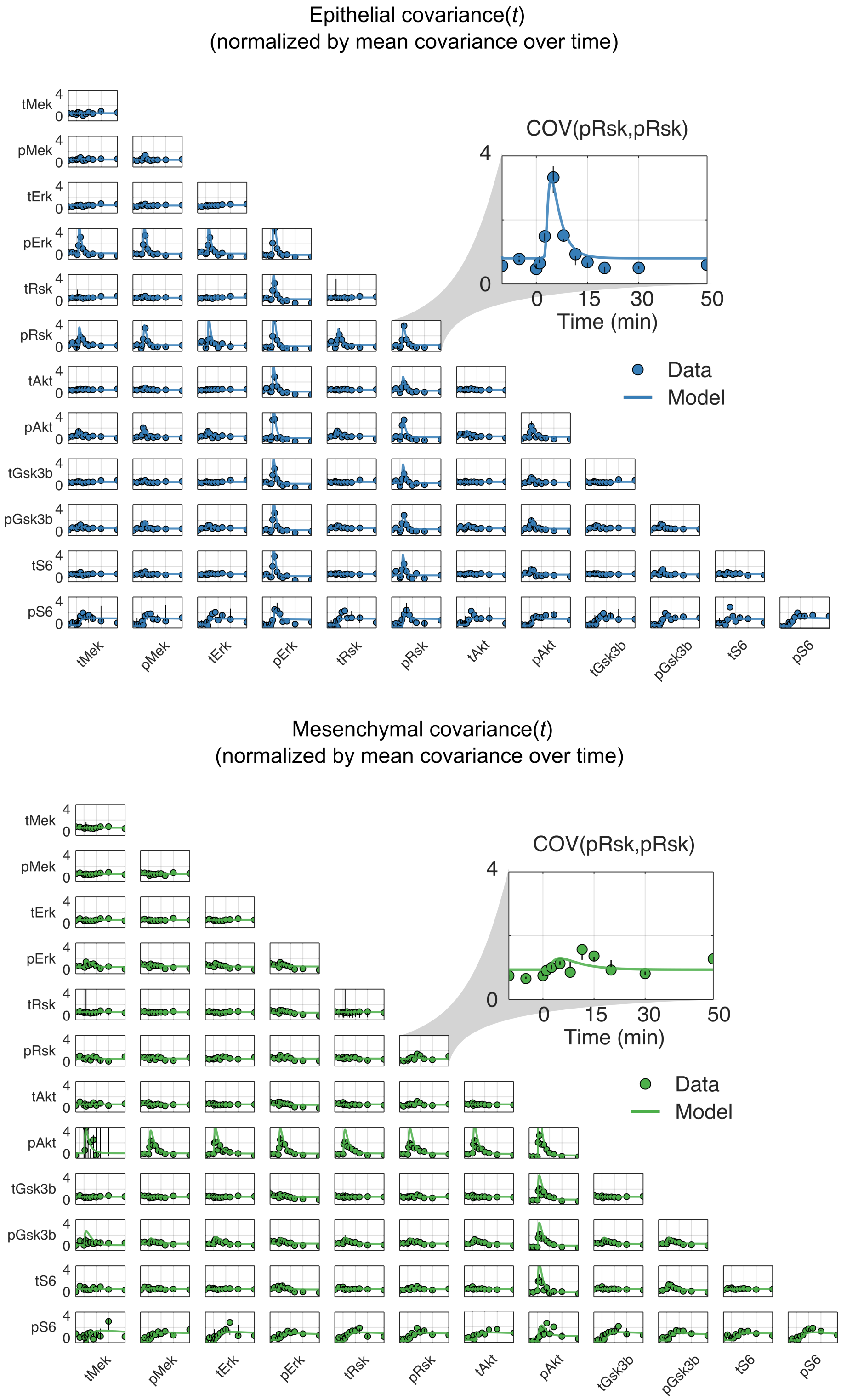

Figure S3: Model and data covariance over time for all total and phosphoprotein pairs. As in Figure 3, including total proteins. Data are given as dots. Colored lines represent model results. Black lines indicate the range of data across replicates.

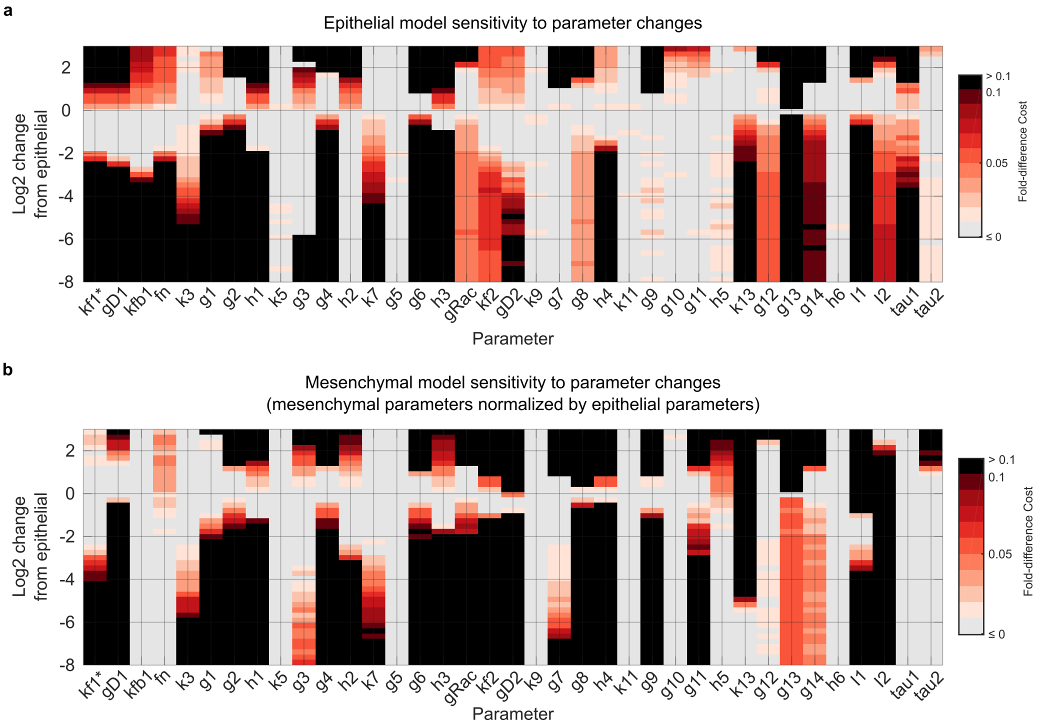

Figure S4: Grid-based sensitivity of the model to both cell phenotypes. (**a**) Model sensitivities calculated using epithelial cells as in Figure 4. (**b**) Model sensitivities calculated using mesenchymal cells. Both plots show parameter values as $\log_2$ fold changes from the optimal parameter values for epithelial cells.

Element	Antibody	Clone	Vendor	[μg/mL]
139La	pCREB	J151-21	BD	1.50
141Pr	pSTAT1	4a	BD	2.00
142Nd	PTEN	138G6	CST	0.75
143Nd	ERK1/2	137F5	CST	1.00
144Nd	pMEK1/2	166F8	CST	1.00
146Nd	pSTAT5	Poly6511	BL	2.00
147Sm	GSK3β	D5C5Z	CST	1.25
148Nd	pS6K	1A5	CST	1.25
149Sm	P90RSK2	D21B2	CST	0.75
150Nd	MEK	D1A5	CST	2.25
151Eu	pEGFR	Y38	Abcam	1.50
152Sm	pAMPKα	40H9	CST	1.50
153Eu	pAKT	D9E	CST	1.00
154Sm	pERK1/2	20A	BD	0.75
155Gd	STAT1	SM1	Abcam	2.00
156Gd	CYCLIN B1	GNS-11	BD	1.00
158Gd	pGSK3	D85E12	CST	0.25
159Tb	GAPDH	6C5	TSPA	0.50
160Gd	mTOR	7C10	CST	1.00
161Dy	pPDPK1	J66-653.44.22	BD	0.05
162Dy	Vimenten	D21H3	CST	0.75
163Dy	pP90RSK	D5D8	CST	1.25
164Dy	AKT	C67E7	CST	0.50
165Ho	non-p-β-CATENIN	D13A1	CST	0.75
166Er	pSTAT3	4/pSTAT3	BD	1.50
167Er	STAT3	124H6	CST	0.75
168Er	pPLCγ2	K86-689.37	BD	1.00
169Tm	EGFR	AY13	BL	1.00
170Er	pHH3	HTA28	BL	0.50
171Yb	pS6	N7-548	BD	0.10
172Yb	cleaved PARP	F21-852	BD	2.00
173Yb	pMTOR	D9C2	CST	2.00
174Yb	E-CADHERIN	36/E	BD	0.50
175Lu	S6	54D2	CST	0.25
176Yb	p4EBP1	236B4	CST	0.25

Table S4: Antibody panel and staining concentrations. Vendor abbreviations: BD Biosciences (BD), BioLegend (BL), Cell Signaling Technologies (CST), Thermo Scientific Pierce Antibodies (TSPA). Measured phosphoproteins that did not show robust dynamics in response to EGF stimulation were omitted from modeling.

Theta$_j$	LB	UB	Space	$\Theta^*_{E,j}$	$\Theta^*_{M,j}$	$\hat{\Theta}_{E^*,j}$	$\hat{\Theta}_{M^*,j}$
k_{f1}	-3	1	log_{10}	-0.3851	-0.3523	-0.3851	-0.3851
g_{d1}	0	6	$Linear$	3.4558	6.6768	3.4558	3.4558
k_{fb1}	-3	2	log_{10}	1.6608	1.6608	1.6608	1.6608
f_n	0	6	$Linear$	1.1531	0.0506	1.1531	1.1531
k_3	-4	1	log_{10}	-2.6751	-2.0672	-2.6751	-2.6751
g_1	0	2	$Linear$	1.1231	1.1231	1.1231	1.1231
g_2	0	2	$Linear$	2.0043	2.0043	2.0043	2.0043
h_1	0	2	$Linear$	1.7707	1.7707	1.7707	1.7707
k_5	-8	0	log_{10}	-2.3842	-3.5491	-2.3842	-2.3842
g_3	0	2	$Linear$	1.4286	1.4286	1.4286	1.4286
g_4	0	6	$Linear$	3.1887	3.1887	3.1887	3.1887
h_2	0	2	$Linear$	0.2818	0.2818	0.2818	0.2818
k_7	-4	2	log_{10}	0.3325	0.3325	0.3325	0.3325
g_5	0	2	$Linear$	0.0049	0.0049	0.0049	0.0049
g_6	0	6	$Linear$	0.8883	0.8883	0.8883	0.8883
h_3	0	2	$Linear$	1.5277	1.5277	1.5277	1.5277
g_{Rac}	0	2	$Linear$	0.2448	0.2448	0.2448	0.2448
k_{f2}	-3	1	log_{10}	-0.0884	-0.1008	-0.0884	-0.0884
g_{d2}	0	6	$Linear$	1.6475	1.3986	1.3986	1.3986
k_9	-4	1	log_{10}	-0.8379	-0.8192	-0.8379	-0.8379
g_7	0	2	$Linear$	3.9439	3.9439	3.9439	3.9439
g_8	0	2	$Linear$	2.9885	2.9885	2.9885	2.9885
h_4	0	2	$Linear$	2.5938	2.5938	2.5938	2.5938
k_{11}	-4	1	log_{10}	0.5874	0.5874	0.5874	0.5874
g_9	0	2	$Linear$	4.0000	4.0000	4.0000	4.0000
g_{10}	0	2	$Linear$	0.2000	0.2000	0.2000	0.2000
g_{11}	0	2	$Linear$	0.2000	0.2000	0.2000	0.2000
h_5	0	2	$Linear$	0.3000	0.0375	0.3000	0.0375
k_{13}	-8	1	log_{10}	-7.7709	-10.0401	-7.7709	-9.8
g_{12}	0	2	$Linear$	0.0848	0.0848	0.0848	0.0848
g_{13}	0	2	$Linear$	4.0318	4.0318	4.0318	4.0318
g_{14}	0	2	$Linear$	0.3492	0.3492	0.3492	0.3492
h_6	-1	2	$Linear$	0.0001	0.0001	0.0001	0.0001
I_1	0	2	log_{10}	0.7223	0.0988	0.7223	0.0988
I_2	0	2	log_{10}	0.9102	1.6990	0.9102	1.6990
τ_1	0	3	$Linear$	2.1413	0.7861	2.1413	2.1413
τ_2	0	3	$Linear$	0.4394	0.4517	0.4394	0.4394

Table S5: Search space and resulting point estimates of ODE model parameter sets. Lower (LB) and upper (UB) bounds and associated space for initial Latin hypercube sampling of each parameter during global search step. Note the local search step was unconstrained and could therefore search beyond these bounds. Parameter point estimates for initial epithelial ($\Theta^*_{E,j}$) and mesenchymal ($\Theta^*_{M,j}$) after local search. Parameter point estimates for epithelial ($\hat{\Theta}_{E^*,j}$) and mesenchymal ($\hat{\Theta}_{M^*,j}$) cells after reconciliation across EMT as described in equation (1).
