# Supplementary material for: Mechanistic Model of Signaling Dynamics Across an Epithelial Mesenchymal Transition
Source: Front Physiol. 2020 Nov 30;11:579117. doi: 10.3389/fphys.2020.579117 (PMC7733964; doi:10.3389/fphys.2020.579117)
Supplement: Supplementary file 2 [file Table_1.DOCX]

% supplements

\documentclass[10pt]{article}

% supplements

\usepackage{cell}

\usepackage{paperStyle}

\myexternaldocument{File1}

\renewcommand{\abstractname}{Introduction}

%% clear header

\fancyhead{}

%\begin{frontmatter}

\title{\singlespacing Supplementary Notes: Mechanistic Model of Signaling Dynamics Across an Epithelial Mesenchymal Transition}

\doublespacing

\author[1,2]{James D. Wade}

\author[2,3]{Xiao-Kang Lun}

\author[2]{Nevena Zivanovic}

\author[1,*,+]{\mbox{Eberhard O. Voit}}

\author[2,*]{\mbox{James Wade, Xiao-Kang Lun, Nevena Zivanovic, Eberhard Voit, Bernd Bodenmiller}}

\date{}

\begin{document}

\maketitle

\thispagestyle{empty}

\doublespacing

\begin{abstract}

\doublespacing

Here, we provide additional details regarding the single-cell ODE model of EGF signaling as presented in the main text. This material includes description of data exclusion and scaling before use in modeling, the mathematical formulation of the model, and supplementary figures and tables.

\end{abstract}

% renew table and figure labels

\renewcommand{\thetable}{S\arabic{table}}

\renewcommand{\thefigure}{S\arabic{figure}}

\setcounter{figure}{0}

\setcounter{table}{0}

\subsection*{Data exclusion criteria}

Antibody labeling and cell staining were optimized to minimize the number of cell events with zero or low ion counts in measurement channels used for modeling. Furthermore, measured cell events with fewer than 5 ion counts in total protein channels were excluded. The choice of five counts was made as a trade-off between the number of discarded events, which increases as the threshold increases, and the uncertainty of single-cell measurements, which is a decreasing function of ion counts. This cutoff excluded fewer than 1\% of cells per sample.

\subsection*{Data scaling for use in modeling}

Mass cytometry provides relative values of protein abundance. Absolute values depend on factors such as antibody labeling efficiency, antibody staining, and detection sensitivity. Although relative differences between measured protein abundances can often be subsumed into reaction parameters, we rescaled measured protein levels by measurement channel. As absolute values of protein abundance were not available for Py2T cells, total protein abundance values were taken to be 500 in arbitrary units. These values were used to estimate the average concentration $\bar{\mathbf{x}}_j$ of each protein $j$ in a cell. Protein measurements were then linearly scaled as follows:

Given a protein $j$, its associated average concentration $\bar{\mathbf{x}}_j$ in a sample and its measured steady-state distribution $D_{\mathbf{x}_{j|ss}}$, a scaling factor $z_j$ was calculated as

%

\begin{linenomath}

\begin{equation}

z_{j} = \frac{\bar{\mathbf{x}}_j}{\textrm{E}\big[D_{\mathbf{x}_{j|ss}}\big]}.

\end{equation}

\end{linenomath}

%

This implementation represents a linear scaling factor of the experimentally measured steady-state distribution of protein $j$ such that the mean of the steady-state distribution $\textrm{E}[D_{\mathbf{x}_{j|ss}}]$ is equal to the population average $\bar{\mathbf{x}}_j$, arbitrarily set at 500. For all experimental measurements, each total protein $j$ was scaled using the corresponding $z_{j}$. In other words, all time points were scaled using a factor calculated at steady state.

Steady-state levels of phosphoproteins clearly cannot be greater than those of the corresponding total protein. Additionally, if steady-state phosphoprotein levels are too high relative to total protein, the relative increase in phosphorylation levels in the model would be capped due to a lack of unphosphorylated protein. To avoid these cases, phosphoprotein distributions were scaled such that the average steady-state value of a phosphoprotein was a fractional value of the total protein level. These additional scaling factors for phophoproteins relative to total proteins were set to 0.06; i.e., initial phosphoprotein levels were, on average, 6\% of total protein. These values were chosen to reduce the number of cells in violation of the ``active cannot be greater than total'' constraint at steady state. In the rare case that individual cells violated this constraint, these cells were excluded from the sample for analysis.

\section*{Model of EGF signaling in the MAPK/ERK cascade}

In this section, we describe the mathematical formulation of our model of EGF signaling in the MAPK/ERK cascade.

\subsection*{State variables}

The model uses eight state variables to describe changes in ERK pathway signaling components.

\clearpage

\begin{linenomath}

\begin{table*}[h]

\centering

\begin{tabular}{@{}ll@{}} \toprule

State variable & Description \\ \midrule

I$_1$ & Input to RAF \\

pRaf & Active RAF \\

Mek & Inactive MEK \\

pMek & Active MEK \\

Erk & Inactive ERK \\

ppErk & Active ERK \\

Rsk & Inactive RSK \\

pRsk & Active RSK \\

I$_2$ & Input to PI3K \\

PI3K & Active PI3K \\

Akt & Inactive AKT \\

pAkt & Active AKT \\

pGsk3b & Inactive GSK3$\beta$ \\

Gsk3b & Active GSK3$\beta$ \\

S6 & Inactive S6 \\

pS6 & Active S6 \\ \bottomrule

\end{tabular}

\caption{ODE model state variables}

\label{table:EMT_Var}

\end{table*}

\end{linenomath}

%

Active and total MEK, ERK, RSK, S6, AKT, and GSK3$\beta$ were measured. After scaling, inactive forms were calculated as $\textrm{Inactive} = \textrm{Total} - \textrm{Active}$. The inputs $I_1$ and $I_2$ and initial values of pRAF and PI3K were assumed to be the same for all cells. These choices were made to reduce prior assumptions. The variation in these components that were not explicitly measured were captured by the single-cell parameters $k_{d1}$, $k_4$, $k_{d2}$, and $k_{10}$, which were obtained from steady-state measurements.

\clearpage

\subsection*{Kinetic parameters}

\begin{linenomath}

\begin{table*}[h]

\centering

\begingroup

\small

\begin{tabular}{@{}ll@{}} \toprule

Parameter & Description \\ \midrule

$k_{f1 } $ & Activation rate constant of pRaf by Input$_1$\\

$g_{d1 } $ & Kinetic order of pRaf inactivation\\

$k_{fb1} $ & Hill function inflection point of negative feedback from ppErk\\

$f_{n } $ & Hill coefficient of negative feedback from ppErk\\

$k_{3 } $ & Activation rate constant of pMek\\

$g_{1 } $ & Kinetic order of pMek activation by Mek\\

$g_{2 } $ & Kinetic order of pMek activation by pRAF\\

$h_{1 } $ & Kinetic order of pMek inactivation by pMek\\

$k_{5 } $ & Activation rate constant of ppErk\\

$g_{3 } $ & Kinetic order of ppErk activation by Erk\\

$g_{4 } $ & Kinetic order of ppErk activation from pMek\\

$h_{2 } $ & Kinetic order of ppErk inactivation from ppErk\\

$k_{7 } $ & Activation rate constant of pRsk\\

$g_{5 } $ & Kinetic order of Rsk activation by Rsk\\

$g_{6 } $ & Kinetic order of Rsk activation by ppErk\\

$h_{3 } $ & Kinetic order of pRsk inactivation by pRsk\\

$g_{Rac} $

$k_{f2 } $ & Activation rate constant of PI3K by Input$_2$\\

$g_{d2 } $ & Degradation rate constant of PI3K signal\\

$k_{9 } $ & Activation rate constant of pAkt\\

$g_{7 } $ & Kinetic order of pAkt activation by Akt\\

$g_{8 } $ & Kinetic order of pAkt activation by PI3K\\

$h_{4 } $ & Kinetic order of pAkt inactivation by pAkt\\

$k_{11 } $ & Inctivation rate constant of Gsk3b\\

$g_{9 } $ & Kinetic order of pGsk3b inactivation by Gsk3b\\

$g_{10 } $ & Kinetic order of pGsk3b inactivation by pRsk crosstalk\\

$g_{11 } $ & Kinetic order of pGsk3b inactivation by pAkt\\

$h_{5 } $ & Kinetic order of pGsk3b activation by pGsk3b\\

$k_{13 } $ & Activation rate constant of pS6\\

$g_{12 } $ & Kinetic order of pS6 activation by S6\\

$g_{13 } $ & Kinetic order of pS6 activation by pRsk\\

$g_{14 } $ & Kinetic order of pS6 activation by pAkt\\

$h_{6 } $ & Kinetic order of pS6 inactivation by pS6\\ \bottomrule

\end{tabular}

\endgroup

\caption{Model parameters}

\label{table:EMT_Par}

\end{table*}

\end{linenomath}

%

The rate constants $\{k_d1,k_4.k_6,k_8,k_{d2},k_{10},k_{12},k_{14}\}$ were collected in $\bm{\Phi_k}$ and computed from the steady-state equations. All other parameters were collected in $\bm{\Theta}$ and, therefore, equal across all cells and used as decision variables in the optimization algorithms.

\subsection*{Inputs}

% \clearpage

\begin{linenomath}

\begin{table*}[h]

\centering

\begin{tabular}{@{}ll@{}} \toprule

Input & Description \\ \midrule

I$_1$ & Input (EGF) to pRaf\\

I$_2$ & Input (EGF) to PI3K\\

I$_{ss}$ & Input at steady state (before EGF addition: $t<\tau$) \\

$\tau_1$ & Time delay between addition of EGF and initial pRaf signaling \\

$\tau_2$ & Time delay between addition of EGF and initial PI3K signaling \\ \bottomrule

\end{tabular}

\caption{Model inputs}

\label{table:EMT_Input}

\end{table*}

\end{linenomath}

%

The ERK/AKT pathway components considered in our model were in a pseudo-steady state at time scale of our experiments (1 hour). Thus, our model must also be at steady state before addition of EGF. We arbitrarily chose the input value of $I_1 = I_2 = I_{ss} = 1$ as the pre-stimulation steady-state inputs to our model. We used the time delays $\tau_1$ and $\tau_2$ to represent the delay between experimental addition of EGF to the medium and the time when the signal reached the ERK and AKT signaling branches, respectively. In other words, $\tau_1$ represents the time it takes for signal to be transmitted by all relevant reactions including receptor-ligand binding, receptor activation, and RAF activation. Similarly, $\tau_2$ represents the time required for PI3K activation. Although the exact values of $\tau_1$ and $\tau_2$ for each cell are undoubtedly variable across the population, as not all cells will encounter the EGF signal at the same moment, we assumed that the delay time ($\tau_1$ and $\tau_2$) was the same for each cell; this maintained the entirely deterministic nature of our model. Thus, in the model for each $i\in\{1,2\}$, if time $t<\tau_i$, then Input $I = I_{ss} = 1$. Once time $t = \tau_i$, then input is reset to represent EGF addition and Input $I = I_i$. At all times $t \ge \tau_i$, input $I_i$ is a dependent variable and determined by solution of the ODE system.

\subsection*{Model equations}

\begin{linenomath}

\begin{align*}

&\dot{I_1} = &- k_{f1}*(I_1-I_{ss})\\

&\dot{pRaf} = & k_{f1}*I_1*PI3K^{g_{Rac}} - k_{d1}*pRaf^{g_{D1}}*\frac{ppErk^{f_n}}{k_{fb1}^{f_n} + ppErk^{f_n}}\\

&\dot{Mek} = &- k_3*Mek^{g_1}*pRaf^{g_2} + k_4*pMek^{h_1}\\

&\dot{pMek} = &k_3*Mek^{g_1}*pRaf^{g_2} - k_4*pMek^{h_1}\\

&\dot{Erk} = &- k_5*Erk^{g_3}*pMek^{g_4} + k_6*ppErk^{h_2}\\

&\dot{ppErk} = &k_5*Erk^{g_3}*pMek^{g_4} - k_6*ppErk^{h_2}\\

&\dot{Rsk} = &- k_7*Rsk^{g_5}*ppErk^{g_6} + k_8*pRsk^{h_3}\\

&\dot{pRsk} = & k_7*Rsk^{g_5}*ppErk^{g_6} - k_8*pRsk^{h_3}\\

&\dot{I_2} = &- k_{f2}*(I_2-I_{ss})\\

&\dot{PI3K} = &k_{f2}*I_2 - k_{d2}*pPI3K^{g_{D2}}\\

&\dot{Akt} = &- k_9*Akt^{g_7}*PI3K^{g_8} + k_{10}*pAkt^{h_4}\\

&\dot{pAkt} = & k_9*Akt^{g_7}*PI3K^{g_8} - k_{10}*pAkt^{h_4}\\

&\dot{Gsk3b} = &- k_{11}*Gsk3b_{g_9}*pRsk^{g_{10}}*pAkt^{g_{11}} + k_{12}*pGsk3b^{h_5}\\

&\dot{pGsk3b} = & k_{11}*Gsk3b_{g_9}*pRsk^{g_{10}}*pAkt^{g_{11}} - k_{12}*pGsk3b^{h_5}\\

&\dot{S6} = &- k_{13}*S6^{g_{12}}*pRsk^{g_{13}}*pAkt^{g_{14}} + k_{14}*pS6^{h_6}\\

&\dot{pS6} = & k_{13}*S6^{g_{12}}*pRsk^{g_{13}}*pAkt^{g_{14}} - k_{14}*pS6^{h_6}\\

\end{align*}

\end{linenomath}

\clearpage

\section*{Supplementary Figures and Tables}

% supp fig 1

% figure 1

\begin{figure}[h]

\centering

\includegraphics[width=0.9\textwidth]{Supp_Figures/Supp_Fig1.png}

\footnotesize

\caption{Microscopy images of TGF-$\beta$ treatment inducing an EMT. At day 0 all cells grow in a monolayer with a cobblestone epithelial morphology. By day 3, some of the cells are no longer constrained to monolayer growth and have transitioned to an elongated mesenchymal morphology.}

\label{fig:EMT_Supp1}

\end{figure}

\clearpage

% supp fig 2

\begin{figure}[h]

\centering

\includegraphics[width=0.9\textwidth]{Supp_Figures/Supp_Fig2.png}

\footnotesize

\caption{Partial correlation of phospho- and total signaling kinases for epithelial and mesenchymal cell populations. (\textbf{a}) Heatmaps of correlations of phospho- and total kinases ordered by clustering of the partial correlations of epithelial cells as shown in the dendrogram. Partial correlations were Fisher z-transformed. (\textbf{b}) Partial correlation values for epithelial cells as in Figure \ref{fig:EMT_figure2}b. The cutoffs for network edges of $\rho \ge |0.1|$ are marked by the red lines. (\textbf{c}) The distribution of partial correlation values for epithelial cells as in Figure \ref{fig:EMT_Supp2}a. Red lines indicate $\rho = |0.1|$.}

\label{fig:EMT_Supp2}

\end{figure}

\clearpage

% supp fig 3

\begin{figure}[h]

\centering

\includegraphics[totalheight=20cm]{Supp_Figures/Supp_Fig3.png}

\footnotesize

\caption{Model and data covariance over time for all total and phosphoprotein pairs. As in Figure \ref{fig:EMT_figure3}, including total proteins. Data are given as dots. Colored lines represent model results. Black lines indicate the range of data across replicates.}

\label{fig:EMT_Supp3}

\end{figure}

\clearpage

% supp fig 4

\begin{figure}[h]

\centering

\includegraphics[width=0.9\textwidth]{Supp_Figures/Supp_Fig4.png}

\footnotesize

\caption{Grid-based sensitivity of the model to both cell phenotypes. (\textbf{a}) Model sensitivities calculated using epithelial cells as in Figure \ref{fig:EMT_figure4}. (\textbf{b}) Model sensitivities calculated using mesenchymal cells. Both plots show parameter values as log$_2$ fold changes from the optimal parameter values for epithelial cells.}

\label{fig:EMT_Supp4}

\end{figure}

\clearpage

% antibody panel

\begin{linenomath}

\begin{table*}[h]

\centering

% \begingroup

% \footnotesize

\begin{tabular}{@{}lllll@{}} \toprule

Element & Antibody & Clone & Vendor & [$\mu$g/mL] \\ \midrule

139La & pCREB & J151-21 & BD & 1.50\\

141Pr & pSTAT1 & 4a & BD & 2.00\\

142Nd & PTEN & 138G6 & CST & 0.75\\

143Nd & ERK1/2 & 137F5 & CST & 1.00\\

144Nd & pMEK1/2 & 166F8 & CST & 1.00\\

146Nd & pSTAT5 & Poly6511 & BL & 2.00\\

147Sm & GSK3$\beta$ & D5C5Z & CST & 1.25\\

148Nd & pS6K & 1A5 & CST & 1.25\\

149Sm & P90RSK2 & D21B2 & CST & 0.75\\

150Nd & MEK & D1A5 & CST & 2.25\\

151Eu & pEGFR & Y38 & Abcam & 1.50\\

152Sm & pAMPK$\alpha$ & 40H9 & CST & 1.50\\

153Eu & pAKT & D9E & CST & 1.00\\

154Sm & pERK1/2 & 20A & BD & 0.75\\

155Gd & STAT1 & SM1 & Abcam & 2.00\\

156Gd & CYCLIN B1 & GNS-11 & BD & 1.00\\

158Gd & pGSK3 & D85E12 & CST & 0.25\\

159Tb & GAPDH & 6C5 & TSPA & 0.50\\

160Gd & mTOR & 7C10 & CST & 1.00\\

161Dy & pPDPK1 & J66-653.44.22 & BD & 0.05\\

162Dy & Vimenten & D21H3 & CST & 0.75\\

163Dy & pP90RSK & D5D8 & CST & 1.25\\

164Dy & AKT & C67E7 & CST & 0.50\\

165Ho & non-p-$\beta$-CATENIN & D13A1 & CST & 0.75\\

166Er & pSTAT3 & 4/pSTAT3 & BD & 1.50\\

167Er & STAT3 & 124H6 & CST & 0.75\\

168Er & pPLC$\gamma$2 & K86-689.37 & BD & 1.00\\

169Tm & EGFR & AY13 & BL & 1.00\\

170Er & pHH3 & HTA28 & BL & 0.50\\

171Yb & pS6 & N7-548 & BD & 0.10\\

172Yb & cleaved PARP & F21-852 & BD & 2.00\\

173Yb & pMTOR & D9C2 & CST & 2.00\\

174Yb & E-CADHERIN & 36/E & BD & 0.50\\

175Lu & S6 & 54D2 & CST & 0.25\\

176Yb & p4EBP1 & 236B4 & CST & 0.25\\ \bottomrule

\end{tabular}

% \endgroup

\caption{Antibody panel and staining concentrations. Vendor abbreviations: BD Biosciences (BD), BioLegend (BL), Cell Signaling Technologies (CST), Thermo Scientific Pierce Antibodies (TSPA). Measured phosphoproteins that did not show robust dynamics in response to EGF stimulation were omitted from modeling.}

\label{table:EMT_Panel}

\end{table*}

\end{linenomath}

\clearpage

\begin{table*}[h!]

\centering

\begin{tabular}{@{}rd{0}d{0}cd{0}d{0}d{0}d{0}@{}} \toprule

Theta$_j$ & \textrm{LB} & \textrm{UB} & Space & \Theta^*_{E,j} & \Theta^*_{M,j} & \hat{\Theta}_{E^*,j} & \hat{\Theta}_{M^*,j}\\ \midrule

$k_{f1 } $ & $ -3 $ & $ 1 $ & $ log_{10} $ & $ -0.3851 $ & $ -0.3523 $ & $ -0.3851 $ & $ -0.3851 $ \\

$g_{d1 } $ & $ 0 $ & $ 6 $ & $ Linear $ & $ 3.4558 $ & $ 6.6768 $ & $ 3.4558 $ & $ 3.4558 $ \\

$k_{fb1} $ & $ -3 $ & $ 2 $ & $ log_{10} $ & $ 1.6608 $ & $ 1.6608 $ & $ 1.6608 $ & $ 1.6608 $ \\

$f_{n } $ & $ 0 $ & $ 6 $ & $ Linear $ & $ 1.1531 $ & $ 0.0506 $ & $ 1.1531 $ & $ 1.1531 $ \\

$k_{3 } $ & $ -4 $ & $ 1 $ & $ log_{10} $ & $ -2.6751 $ & $ -2.0672 $ & $ -2.6751 $ & $ -2.6751 $ \\

$g_{1 } $ & $ 0 $ & $ 2 $ & $ Linear $ & $ 1.1231 $ & $ 1.1231 $ & $ 1.1231 $ & $ 1.1231 $ \\

$g_{2 } $ & $ 0 $ & $ 2 $ & $ Linear $ & $ 2.0043 $ & $ 2.0043 $ & $ 2.0043 $ & $ 2.0043 $ \\

$h_{1 } $ & $ 0 $ & $ 2 $ & $ Linear $ & $ 1.7707 $ & $ 1.7707 $ & $ 1.7707 $ & $ 1.7707 $ \\

$k_{5 } $ & $ -8 $ & $ 0 $ & $ log_{10} $ & $ -2.3842 $ & $ -3.5491 $ & $ -2.3842 $ & $ -2.3842 $ \\

$g_{3 } $ & $ 0 $ & $ 2 $ & $ Linear $ & $ 1.4286 $ & $ 1.4286 $ & $ 1.4286 $ & $ 1.4286 $ \\

$g_{4 } $ & $ 0 $ & $ 6 $ & $ Linear $ & $ 3.1887 $ & $ 3.1887 $ & $ 3.1887 $ & $ 3.1887 $ \\

$h_{2 } $ & $ 0 $ & $ 2 $ & $ Linear $ & $ 0.2818 $ & $ 0.2818 $ & $ 0.2818 $ & $ 0.2818 $ \\

$k_{7 } $ & $ -4 $ & $ 2 $ & $ log_{10} $ & $ 0.3325 $ & $ 0.3325 $ & $ 0.3325 $ & $ 0.3325 $ \\

$g_{5 } $ & $ 0 $ & $ 2 $ & $ Linear $ & $ 0.0049 $ & $ 0.0049 $ & $ 0.0049 $ & $ 0.0049 $ \\

$g_{6 } $ & $ 0 $ & $ 6 $ & $ Linear $ & $ 0.8883 $ & $ 0.8883 $ & $ 0.8883 $ & $ 0.8883 $ \\

$h_{3 } $ & $ 0 $ & $ 2 $ & $ Linear $ & $ 1.5277 $ & $ 1.5277 $ & $ 1.5277 $ & $ 1.5277 $ \\

$g_{Rac} $ & $ 0 $ & $ 2 $ & $ Linear $ & $ 0.2448 $ & $ 0.2448 $ & $ 0.2448 $ & $ 0.2448 $ \\

$k_{f2 } $ & $ -3 $ & $ 1 $ & $ log_{10} $ & $ -0.0884 $ & $ -0.1008 $ & $ -0.0884 $ & $ -0.0884 $ \\

$g_{d2 } $ & $ 0 $ & $ 6 $ & $ Linear $ & $ 1.6475 $ & $ 1.3986 $ & $ 1.3986 $ & $ 1.3986 $ \\

$k_{9 } $ & $ -4 $ & $ 1 $ & $ log_{10} $ & $ -0.8379 $ & $ -0.8192 $ & $ -0.8379 $ & $ -0.8379 $ \\

$g_{7 } $ & $ 0 $ & $ 2 $ & $ Linear $ & $ 3.9439 $ & $ 3.9439 $ & $ 3.9439 $ & $ 3.9439 $ \\

$g_{8 } $ & $ 0 $ & $ 2 $ & $ Linear $ & $ 2.9885 $ & $ 2.9885 $ & $ 2.9885 $ & $ 2.9885 $ \\

$h_{4 } $ & $ 0 $ & $ 2 $ & $ Linear $ & $ 2.5938 $ & $ 2.5938 $ & $ 2.5938 $ & $ 2.5938 $ \\

$k_{11 } $ & $ -4 $ & $ 1 $ & $ log_{10} $ & $ 0.5874 $ & $ 0.5874 $ & $ 0.5874 $ & $ 0.5874 $ \\

$g_{9 } $ & $ 0 $ & $ 2 $ & $ Linear $ & $ 4.0000 $ & $ 4.0000 $ & $ 4.0000 $ & $ 4.0000 $ \\

$g_{10 } $ & $ 0 $ & $ 2 $ & $ Linear $ & $ 0.2000 $ & $ 0.2000 $ & $ 0.2000 $ & $ 0.2000 $ \\

$g_{11 } $ & $ 0 $ & $ 2 $ & $ Linear $ & $ 0.2000 $ & $ 0.2000 $ & $ 0.2000 $ & $ 0.2000 $ \\

$h_{5 } $ & $ 0 $ & $ 2 $ & $ Linear $ & $ 0.3000 $ & $ 0.0375 $ & $ 0.3000 $ & $ 0.0375 $ \\

$k_{13 } $ & $ -8 $ & $ 1 $ & $ log_{10} $ & $ -7.7709 $ & $ -10.0401 $ & $ -7.7709 $ & $ -9.8 $ \\

$g_{12 } $ & $ 0 $ & $ 2 $ & $ Linear $ & $ 0.0848 $ & $ 0.0848 $ & $ 0.0848 $ & $ 0.0848 $ \\

$g_{13 } $ & $ 0 $ & $ 2 $ & $ Linear $ & $ 4.0318 $ & $ 4.0318 $ & $ 4.0318 $ & $ 4.0318 $ \\

$g_{14 } $ & $ 0 $ & $ 2 $ & $ Linear $ & $ 0.3492 $ & $ 0.3492 $ & $ 0.3492 $ & $ 0.3492 $ \\

$h_{6 } $ & $ -1 $ & $ 2 $ & $ Linear $ & $ 0.0001 $ & $ 0.0001 $ & $ 0.0001 $ & $ 0.0001 $ \\

$I_{1 } $ & $ 0 $ & $ 2 $ & $ log_{10} $ & $ 0.7223 $ & $ 0.0988 $ & $ 0.7223 $ & $ 0.0988 $ \\

$I_{2 } $ & $ 0 $ & $ 2 $ & $ log_{10} $ & $ 0.9102 $ & $ 1.6990 $ & $ 0.9102 $ & $ 1.6990 $ \\

$\tau_1 $ & $ 0 $ & $ 3 $ & $ Linear $ & $ 2.1413 $ & $ 0.7861 $ & $ 2.1413 $ & $ 2.1413 $ \\

$\tau_2 $ & $ 0 $ & $ 3 $ & $ Linear $ & $ 0.4394 $ & $ 0.4517 $ & $ 0.4394 $ & $ 0.4394 $ \\ \bottomrule

\end{tabular}

\caption{Search space and resulting point estimates of ODE model parameter sets. Lower (LB) and upper (UB) bounds and associated space for initial Latin hypercube sampling of each parameter during global search step. Note the local search step was unconstrained and could therefore search beyond these bounds. Parameter point estimates for initial epithelial ($\Theta^*_{E,j}$) and mesenchymal ($\Theta^*_{M,j}$) after local search. Parameter point estimates for epithelial ($\hat{\Theta}_{E^*,j}$) and mesenchymal ($\hat{\Theta}_{M^*,j}$) cells after reconciliation across EMT as described in equation (\ref{eq:EMT}).}

\label{tab:ThetaBoundsResults}

\end{table*}

\end{document}
